# DNA methylation signature of human fetal alcohol spectrum disorder

**DOI:** 10.1186/s13072-016-0074-4

**Published:** 2016-06-29

**Authors:** Elodie Portales-Casamar, Alexandre A. Lussier, Meaghan J. Jones, Julia L. MacIsaac, Rachel D. Edgar, Sarah M. Mah, Amina Barhdadi, Sylvie Provost, Louis-Philippe Lemieux-Perreault, Max S. Cynader, Albert E. Chudley, Marie-Pierre Dubé, James N. Reynolds, Paul Pavlidis, Michael S. Kobor

**Affiliations:** Centre for High-Throughput Biology, University of British Columbia, Vancouver, BC Canada; Department of Medical Genetics, Centre for Molecular Medicine and Therapeutics, Child and Family Research Institute, University of British Columbia, Vancouver, BC Canada; Beaulieu-Saucier Pharmacogenomics Centre, Montreal Heart Institute, Université de Montréal, Montreal, QC Canada; Brain Research Centre, University of British Columbia, Vancouver, BC Canada; Department of Pediatrics and Child Health, Faculty of Medicine, University of Manitoba, Winnipeg, MB Canada; Department of Biochemistry and Medical Genetics, Faculty of Medicine, University of Manitoba, Winnipeg, MB Canada; Faculty of Medicine, Université de Montréal, Montreal, QC Canada; Centre for Neuroscience Studies, Queen’s University, Kingston, ON Canada; Human Early Learning Partnership, School of Population and Public Health, University of British Columbia, Vancouver, British Columbia Canada

## Abstract

**Background:**

Prenatal alcohol exposure is the leading preventable cause of behavioral and cognitive deficits, which may affect between 2 and 5 % of children in North America. While the underlying mechanisms of alcohol’s effects on development remain relatively unknown, emerging evidence implicates epigenetic mechanisms in mediating the range of symptoms observed in children with fetal alcohol spectrum disorder (FASD). Thus, we investigated the effects of prenatal alcohol exposure on genome-wide DNA methylation in the NeuroDevNet FASD cohort, the largest cohort of human FASD samples to date.

**Methods:**

Genome-wide DNA methylation patterns of buccal epithelial cells (BECs) were analyzed using the Illumina HumanMethylation450 array in a Canadian cohort of 206 children (110 FASD and 96 controls). Genotyping was performed in parallel using the Infinium HumanOmni2.5-Quad v1.0 BeadChip.

**Results:**

After correcting for the effects of genetic background, we found 658 significantly differentially methylated sites between FASD cases and controls, with 41 displaying differences in percent methylation change >5 %. Furthermore, 101 differentially methylated regions containing two or more CpGs were also identified, overlapping with 95 different genes. The majority of differentially methylated genes were highly expressed at the level of mRNA in brain samples from the Allen Brain Atlas, and independent DNA methylation data from cortical brain samples showed high correlations with BEC DNA methylation patterns. Finally, overrepresentation analysis of genes with up-methylated CpGs revealed a significant enrichment for neurodevelopmental processes and diseases, such as anxiety, epilepsy, and autism spectrum disorders.

**Conclusions:**

These findings suggested that prenatal alcohol exposure is associated with distinct DNA methylation patterns in children and adolescents, raising the possibility of an epigenetic biomarker of FASD.

**Electronic supplementary material:**

The online version of this article (doi:10.1186/s13072-016-0074-4) contains supplementary material, which is available to authorized users.

## Background

The prenatal environment has the potential to permanently imprint physiological and behavioral systems during development, leading to both short- and long-term health consequences. In particular, prenatal alcohol exposure (PAE) can alter the development, function, and regulation of numerous neural and physiological systems, resulting in a variety of deficits falling under the umbrella of fetal alcohol spectrum disorder (FASD) [1]. Over the lifetime, the effects of PAE are manifested through cognitive and behavioral deficits, persistent alterations to stress responsivity and immune function, and increased vulnerability to mental health disorders and other comorbidities in individuals with FASD [1–4]. However, the degree to which alcohol exposure causes alterations during development varies, depending on factors such as timing and level of exposure, overall maternal health and nutrition, and genetic background [5]. As such, only a small proportion of affected children present with the phenotype of fetal alcohol syndrome (FAS), which is distinguished by growth deficits and facial dysmorphisms in addition to central nervous system dysfunction [6, 7]. Nevertheless, the vast majority of children with FASD display physiological and neurobehavioral impairments lasting into adulthood, suggesting persistent programming effects of PAE across the spectrum of FASD [8].

While the etiology of the FASD currently remains unclear, epigenetics is emerging as an attractive candidate for the biological embedding of prenatal and early-life experiences in general and thus is a promising avenue for the study of FASD [9]. Epigenetics refers to modifications of DNA and its packaging that alter the accessibility of DNA to potentially regulate gene expression and cellular function without changes to the underlying genomic sequences [10]. The most studied epigenetic modification in human populations is DNA methylation, which refers to the covalent attachment of a methyl group to the 5′ position of cytosine, typically occurring in the context of cytosine–guanine dinucleotide (CpG) sites [11]. CpG sites are relatively rare in the human genome, yet do not occur at random; regions containing higher than expected levels of these dinucleotides have been termed “CpG islands” (CGIs) [12]. The 2-kb regions flanking CGIs are known as CGI “shores,” while the areas located beyond shores are known as “shelves” [13–15]. Of note, these regions are typically more variable than CGIs themselves, as they have a greater range of DNA methylation across individuals [14]. DNA methylation is associated with the regulation of gene expression, although its effects on transcription are highly dependent on genomic context. For example, when located within gene promoters, DNA methylation generally represses gene expression, but this relationship is less well defined for CpGs located within gene bodies and intergenic regions [16]. Furthermore, DNA methylation is closely associated with several key developmental processes, including genomic imprinting, tissue specification and differentiation [17, 18]. DNA methylation patterns are also population specific, as many CpG sites are associated with ethnicity [19–21]. There are a number of possible reasons for this association, including shared environments or associations of epigenetic marks with specific genetic variants [22–24].

Importantly, DNA methylation is malleable in response to environmental factors, and these changes may be inherited through cell divisions, potentially persisting throughout the lifetime [25–27]. For example, prenatal exposure to cigarette smoke is associated with long-term changes in DNA methylation of the *AHRR* gene, and maternal undernutrition during pregnancy leads to altered DNA methylation of *IGF2* [28, 29]. Several studies have also characterized epigenetic changes following prenatal and postnatal ethanol exposure [30–36]. Early work in pregnant mice demonstrated that acute ethanol exposure during mid-gestation (gestational days 9–11) causes global genomic loss of DNA methylation in the fetus [37]. However, recent studies of embryonic cultures exposed to ethanol show that rather than a global demethylation of the genome by ethanol, some regions become more methylated and others less methylated [38]. Moreover, genome-wide studies in adult mice that were exposed to ethanol prenatally have also identified widespread changes in DNA methylation patterns in the entire brain, further suggesting an important role for epigenetics in the etiology of FASD [39]. Finally, a recent study characterized the DNA methylation profile in buccal epithelial cells (BECs) from a small cohort of human FASD samples, identifying alterations in the epigenome of children with FASD, particularly within the protocadherin gene clusters [40].

Collectively, these findings support epigenetic mechanisms as potential contributors to the deficits observed following PAE. However, no large-scale investigations of DNA methylation in individuals with FASD have been performed to date. In order to ascertain the effect of PAE on the human epigenome, the present study investigated the DNA methylation patterns of BECs from 110 children with FASD and 96 age- and sex-matched controls, to our knowledge representing the largest investigation on PAE effects on the human epigenome. Statistically significant alterations between FASD cases and controls were successfully identified following ethnic background correction, with a number of differentially methylated sites and regions located in genes previously associated with alcohol exposure [38, 40]. Taken together, these results support a potential role for DNA methylation in the etiology of the neurobiological deficits observed in children with FASD and represent a potential epigenetic signature of FASD.

## Results

### The NeuroDevNet FASD epigenetics cohort

Participants in the NeuroDevNet Canadian FASD study cohort were recruited from six clinical sites across Canada (Vancouver, BC; Edmonton, AB; Cold Lake, AB; Winnipeg, MB; Ottawa, ON; and Kingston, ON) [39]. More specifically, 110 children with FASD or confirmed PAE and 96 typically developing controls were matched for sex and age, ranging from 5 to 18 years of age, for the analysis of genome-wide DNA methylation patterns (Table 1). We note that self-declared ethnicity differed considerably between the FASD and control participants, necessitating stringent statistical corrections, as described below.

**Table 1:** Characteristics of the NeuroDevNet FASD cohort

	FASD cases	Controls
*N*	110	96
Age	11.55 ± 3.37	11.28 ± 3.38
Sex		
Male (%)	41	47
Female (%)	59	53
Self-declared ethnicity		
Caucasian	27 % (48 %)^a^	91 % (96 %)
Other	73 % (52 %)	9 % (4 %)

^a^Percentages in brackets include participants with mixed ethnicity including Caucasian

### Children with FASD displayed altered DNA methylation patterns

The DNA methylation profiles of BECs from the complete NeuroDevNet cohort were assessed using the Illumina HumanMethylation450 array, which assays DNA methylation at 485,512 sites across the human genome. Following quality control and normalization to remove probes with bad detection *p*-values and low bead counts, or those associated with sex chromosomes, SNPs, and polymorphic CpGs, 404,430 sites remained in the final dataset of 206 samples [42]. Although BECs typically represent a relatively homogenous population of cells, they can occasionally be contaminated by white blood cells during collection, thus possibly affecting the results of differential DNA methylation analyses [43]. To assess whether BEC from the present study had high levels of contamination, principal component analysis of BECs and blood samples obtained from GEO (GSE42861) was performed. This analysis did not reveal any blood contamination in our dataset, as evidenced by the distant clustering of samples from both tissue types (Additional file 1: Figure S1). Having thus established that cellular heterogeneity was unlikely to confound our results, we next set out to identify alterations in DNA methylation patterns specific to the FASD group. For this, differential DNA methylation analysis using a two-group design was coupled with surrogate variable analysis (SVA), which corrects for batch effects and any other undesirable variation in the data. This analysis identified 1661 differentially methylated (DM) CpG sites between the FASD group and controls at a false discovery rate (FDR) < 0.05, indicating substantial differences in DNA methylation patterns between the two groups. However, self-declared ethnicity in the cohort was strongly confounded with FASD status (Table 1). Given that ethnicity has been associated with altered DNA methylation levels, these differences could potentially drive alterations in DNA methylation at these 1661 DM CpG sites [19–21].

### Ethnic background correction identified FASD-specific DNA methylation patterns

**Fig. 1:**
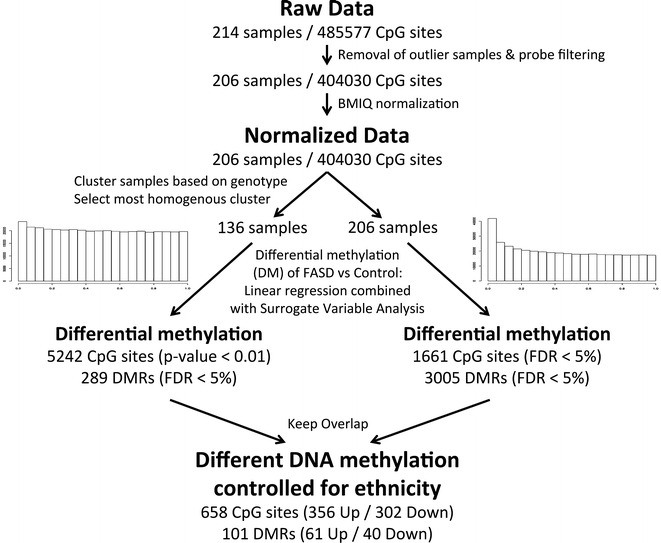
Flowchart of bioinformatic analyses. Two analyses were performed in parallel to assess differential DNA methylation between FASD cases and controls. The first analysis, using 206 samples (110 FASD and 96 controls), identified 1661 differentially methylated (DM) sites and 3005 differentially methylated regions (DMR). The second, using a more genetically homogenous subgroup composed of 49 FASD cases and 87 controls, identified 5242 DM sites and 289 DMRs. These were used to filter out the sites identified in the first analysis that might have been confounded by differences in ethnic proportions between the two groups, resulting in a final list of 658 DM CpGs and 101 DMRs free of the confounding effects of ethnicity

**Fig. 2:**
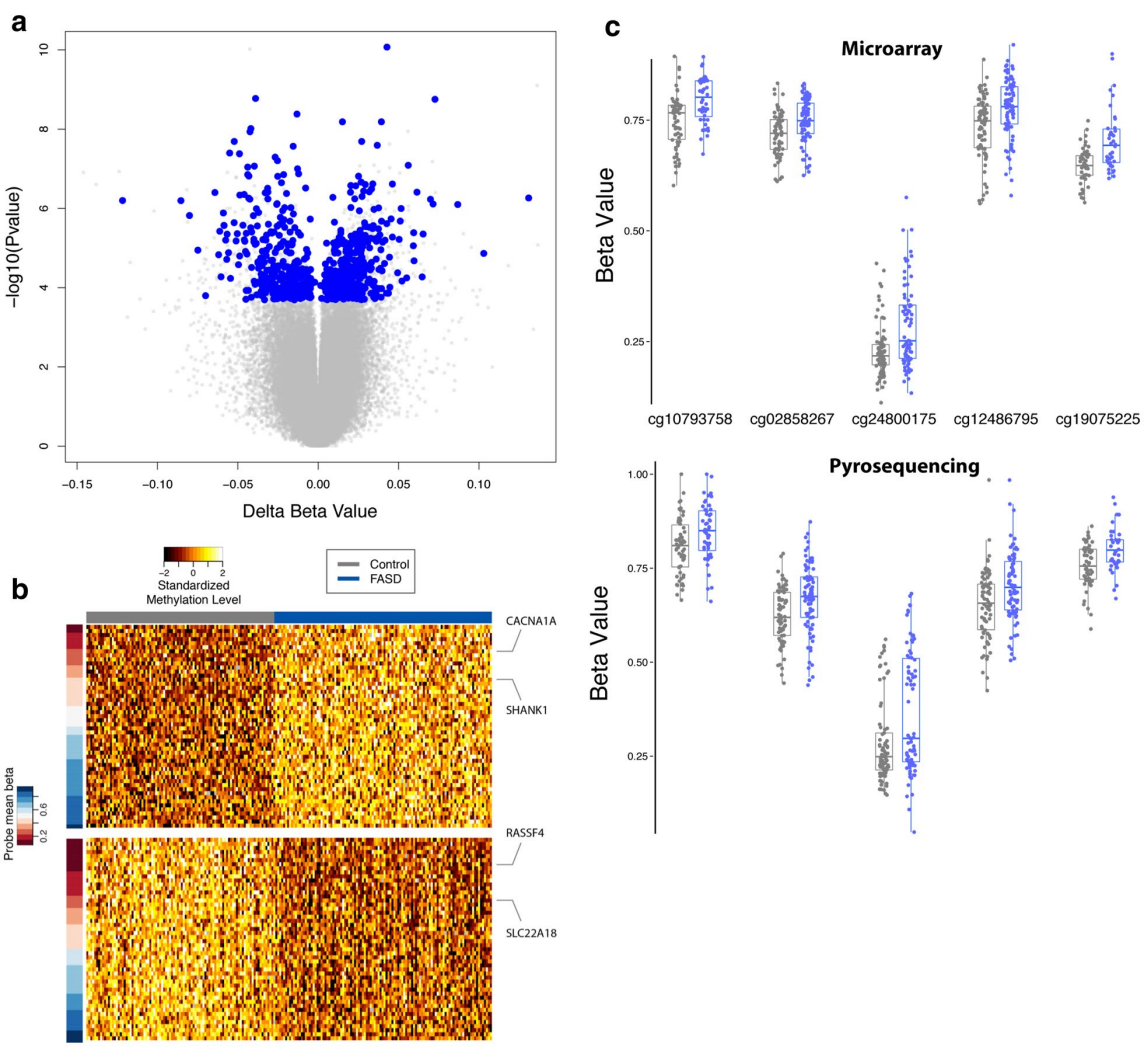
Visualization and verification of differentially methylated probes. **a** Volcano plot showing mean methylation differences between FASD and control (*x* axis) versus log transformed *p*-values (*y* axis). 1661 CpG sites with an FDR <0.05 were considered significantly differently methylated between FASD and control, but 1003 of these were ethnically confounded, resulting in the final 658 probes shown in *blue*. **b** Heatmap of top 50 most significant up- (*top*) and down-methylated (*bottom*) probes in control (*left*, *gray*) versus FASD cases (*right*, *blue*). The percent methylation values (ranging from 0 to 1) are adjusted for the covariates from the regression model and then centered, scaled, and trimmed, resulting in a standardized DNA methylation level ranging from −2 to +2 (*black*–*white* scale). The mean percent methylation value (beta) for each probe (*red*–*blue* scale) is the mean methylation value, after adjustment for covariates, for all samples. **c** Verification with pyrosequencing in both FASD (*blue*) and control (*gray*) samples. The *top panel* displays DNA methylation levels measured by the 450K array, the *bottom panel*, the levels for the same CpG sites measured with pyrosequencing. These CpGs were located in the gene body of *SHANK3* (cg10793758), *NOS1AP* (cg02858267), *CACNA1A* (cg24800175), and *SNED1* (cg19075225), or in the 3′ UTR of *NOS1AP* (cg12486795). Those found in *NOS1AP* were located in a CpG island, while those in *SHANK3* and *CACNA1A* were located in a north shelf or shore, respectively. The CpG associated with *SNED1* was not located near any CpG island

To account for ethnicity on a genetic basis, the Illumina HumanOmni2.5 array was used to obtain genotypes at nearly 2.4 million single nucleotide polymorphisms (SNPs) for each child. Participants were clustered by multi-dimensional scaling (MDS) of genotypic data along with publicly accessible data from the HapMap project [44]. Linear regression of the first four genetic clusters from this analysis with the SVs revealed little correlation with the majority of DNA methylation variation, suggesting that further correction for differences in ethnicity was required to isolate the effects of PAE beyond ethnicity (Additional file 1: Figure S2). As such, individuals clustering within the larger and more genetically homogeneous subgroup were selected for further analysis, consisting of 49 FASD cases and 87 controls (Table 2; Additional file 1: Figure S3; Additional file 3: Table S2). Differential DNA methylation analysis was performed on the more genetically homogeneous subsample to isolate the effects of PAE in the absence of an ethnic confound. In support of less ethnicity-related effects in this subsample, SVA identified fewer SVs compared to the full dataset. Furthermore, the results from DNA methylation analysis in this subgroup displayed only a moderate correlation with those obtained from the full sample (Spearman rank correlation: 0.43), suggesting that ethnicity indeed may have influenced differential DNA methylation patterns in the full cohort, despite our efforts to use SVA to remove the effects of ethnicity. Therefore, the subsample was used to filter out ethnically confounded CpG loci to obtain a subset of DM sites unbiased for ethnicity (Fig. 1). More specifically, the top 5242 probes (unadjusted *p*-value <0.01) in the genetically homogeneous subsample were selected as a conservative set of differentially methylated CpG sites between FASD cases and controls that were unaffected by ethnic background. This set was compared to the 1661 DM sites identified in the full sample, and only the probes present in both lists were considered specific effects of FASD, unlikely to be related to effects of ethnicity. Following this strategy, a final list of 658 DM CpG sites significantly altered in FASD cases was obtained at an FDR < 0.05 (Additional file 2: Table S1), composed of 356 down-methylated and 302 up-methylated sites compared to controls (Fig. 2a, b). To determine whether this corrective analysis removed some or all effects of ethnicity, differential DNA methylation analysis was performed on FASD cases from the two main ethnic clusters from MDS to tease apart ethnicity and FASD-specific effects between the groups (Additional file 1: Supplemental methods). As expected, the ethnicity-corrected CpGs were less associated with ethnic differences in DNA methylation patterns than the uncorrected set of CpGs, as evidenced by the decreased area under the ROC curve (Additional file 1: Figure S4). Furthermore, reflecting the economic realities of our study populations, socioeconomic status (SES) scores were slightly confounded between groups (*p* = 0.00017; Additional file 1: Figure S5), with the FASD group displaying lower overall scores than controls. However, the more ethnically homogeneous subgroup showed less skewing toward low SES in the FASD group (*p* = 0.16; Additional file 1: Figure S5), suggesting that the effects of SES might also have been partially accounted for during the correction for ethnic biases between groups. As such, the ethnicity-corrected set of 658 CpG loci associated with FASD was used in all subsequent analyses. The changes observed in the absolute methylation levels of these DM CpGs were relatively small, consistent with previous human studies of neurological and neurodevelopmental disorders, with percent methylation changes ranging from 0.16 to 13.1 % after correction for surrogate variables (SVs) [45]. However, 41 DM sites passed an arbitrary threshold for possible biological relevance of >5 % difference in DNA methylation levels between groups. Taken together, these results support the hypothesis that FASD is associated with altered DNA methylation patterns, largely free of identified confounding effects due to ethnicity and SES.

**Table 2:** Characteristics of the more genetically homogenous subsample

	FASD cases	Controls
*N*	49	87
Age	11.29 ± 3.16	11.29 ± 3.37
Sex		
Male (%)	43	41
Female (%)	57	59
Self-declared ethnicity		
Caucasian	51 % (76 %)^a^	93 % (97 %)
Other	49 % (24 %)	7 % (3 %)

^a^Percentages in brackets include participants with mixed ethnicity including Caucasian

### Technical verification of FASD DM loci by bisulfite pyrosequencing

To ensure that the results from the differential DNA methylation analysis were not dependent on the method used to measure them, five CpG sites with a difference in percent methylation change >5 % in the vicinity of genes with potential biological relevance were selected for verification using bisulfite pyrosequencing on the same samples. Pyrosequencing results confirmed the DNA methylation levels observed on the 450K array, showing similar DNA methylation levels and differences between groups for CpGs located in *SHANK3*, *NOS1AP*, *CACNA1A*, and *SNED1* (Fig. 2c). Pearson correlations ranged from 0.421 to 0.801 and Bland–Altman plots showed little difference when comparing both methods, suggesting a strong concordance between DNA methylation data from microarray and the different pyrosequencing methods (Additional file 1: Figure S6). Perhaps more importantly, linear regression analysis of pyrosequencing data confirmed differential DNA methylation between FASD cases and controls in this subset of biologically relevant sites, even in the absence of covariates, as the *p*-values ranged from 3.7E−04 to 5.5E−03. Collectively, pyrosequencing data verified the findings from the 450K array, suggesting that individuals with FASD had altered DNA methylation patterns compared to typically developing children.

### Overlap of BEC FASD signatures with brain tissue gene expression and DNA methylation

As alterations to DNA methylation patterns in children with FASD were identified in BECs, it is important to note that changes in peripheral tissues do not necessarily reflect alterations in a relevant tissue, such as the brain, even though these two tissues originate from the same germ layer and thus might share some epigenetic concordance [46]. Therefore, two complimentary approaches were used to obtain an approximation for the relationship of these FASD-associated DM loci to brain biology and possible the etiology of FASD. First, DM genes were compared to publically available gene expression data from 896 postmortem brain regions (Allen Institute for Brain Science) to determine whether they were expressed at biologically relevant levels in neural tissue [47]. This analysis revealed that 56 % of DM genes identified in BECs displayed mRNA expression levels in the brain above the median expression for all genes, with 68 % ranked in the top 2/3 of the genes based on mean ranking across ~900 brain regions [48]. These findings held true whether all DM genes or only the down-/up-methylated genes were considered for analysis. Next, the FASD BEC DNA methylation patterns were compared to DNA methylation patterns from unrelated postmortem cortical brain specimens previously published by our group [48]. The overall correlation of mean DNA methylation between BEC and brain samples for all 658 DM CpGs was 0.76 (Additional file 1: Figure S7). Taken together, these results indicated that BEC may be a suitable surrogate tissue for brain cells and that the DM loci presented here could potentially report on biological alterations in neural tissues.

### FASD DM loci were enriched in regions of high DNA methylation variability

**Fig. 3:**
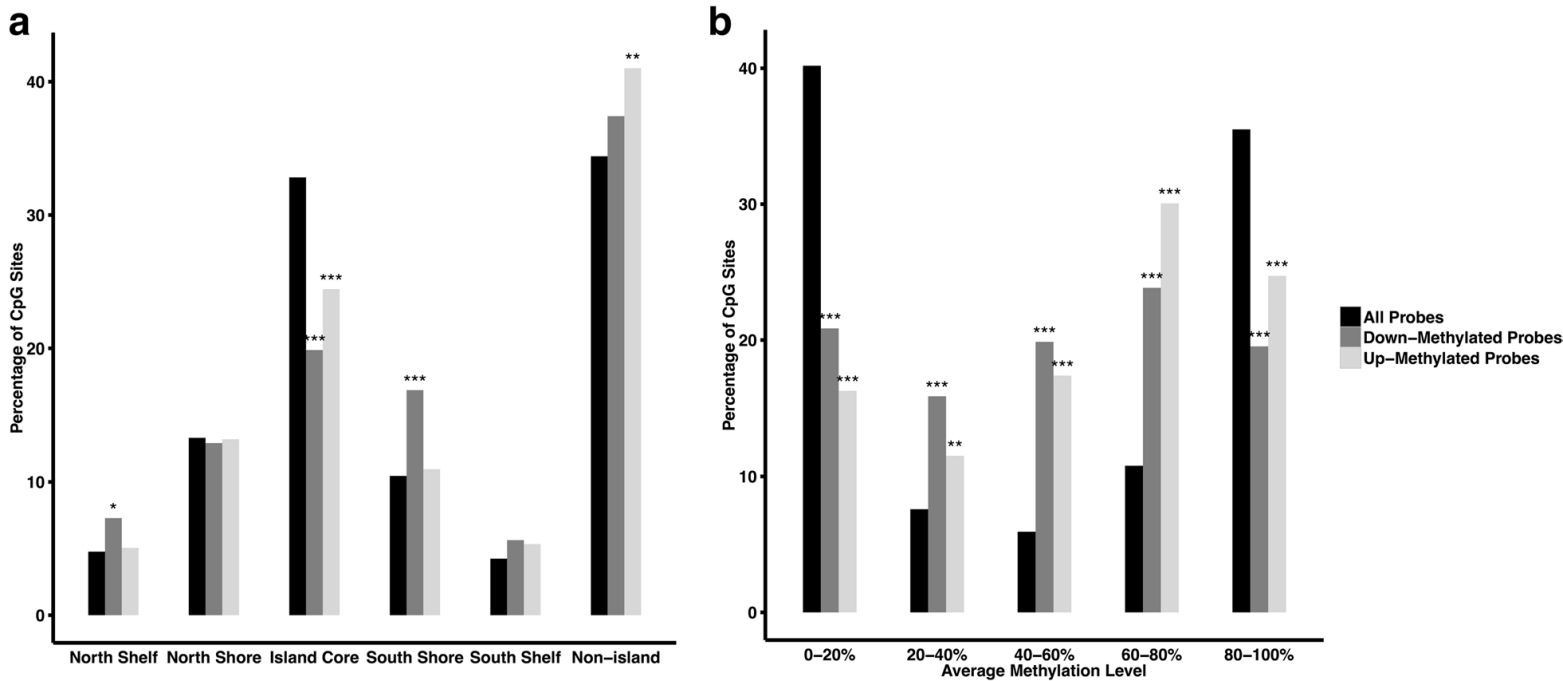
Differentially methylated probes are located in regions of variable and intermediate DNA methylation. **a** The 658 probes differentially methylated between FASD and control were underrepresented in CGI cores (down-methylated *p* = 1.62E−6; up-methylated *p* = 7.53E−4), while down-methylated probes were overrepresented in CGI shores/shelves (*p* = 0.04; *p* = 0.0003) and up-methylated probes were overrepresented in non-CpG island regions (*p* = 0.009). **b** The same probes’ average methylation levels are overrepresented in the mid-range categories (**p < 0.01, ***p < 0.0001)

Given that genomic location plays an important role in sculpting DNA methylation landscapes and mediating its effects, we ascertained the relative enrichment of FASD DM loci in distinct genomic features. Overall, DM probes had a significantly different distribution than the proportions present on the entire 450K array (Fig. 3a; down-methylated probes: *χ*^2^ = 33.63, *p* = 2.8E−06; up-methylated probes: *χ*^2^ = 13.30, *p* = 2.1E−02). Compared to all 450K probes, both down- and up-methylated CpGs in FASD cases were significantly underrepresented in CpG island cores, which generally show the least amount of variability in DNA methylation levels (down-methylated *p* = 1.62E−6; up-methylated *p* = 7.53E−4). By contrast, down-methylated sites were enriched in CpG island shores and shelves (*p* = 0.04; *p* = 0.0003), which tend to be more variable than CpG island cores [14]. Up-methylated sites were overrepresented in non-CpG island regions (*p* = 0.009), further supporting a greater effect of PAE on malleable regions of the epigenome. Moreover, the distribution of average methylation levels for DM sites was significantly different than that of all 404,030 sites (Student’s *t* test; *p* = 2.5E−09; Additional file 1: Figure S8). Further analysis of this phenomenon revealed a significant enrichment for DM CpG sites in the intermediate 20–80 % range of methylation levels, while showing a concordant underrepresentation in the hypo-methylated (<20 %) and hyper-methylated (>80 %) categories (Fig. 3b) [49]. These findings suggested that DM loci in the FASD cases versus controls were mostly located in more variable regions of the epigenome.

### Multiple DM sites were associated with imprinted genes and the protocadherin gene cluster

**Fig. 4:**
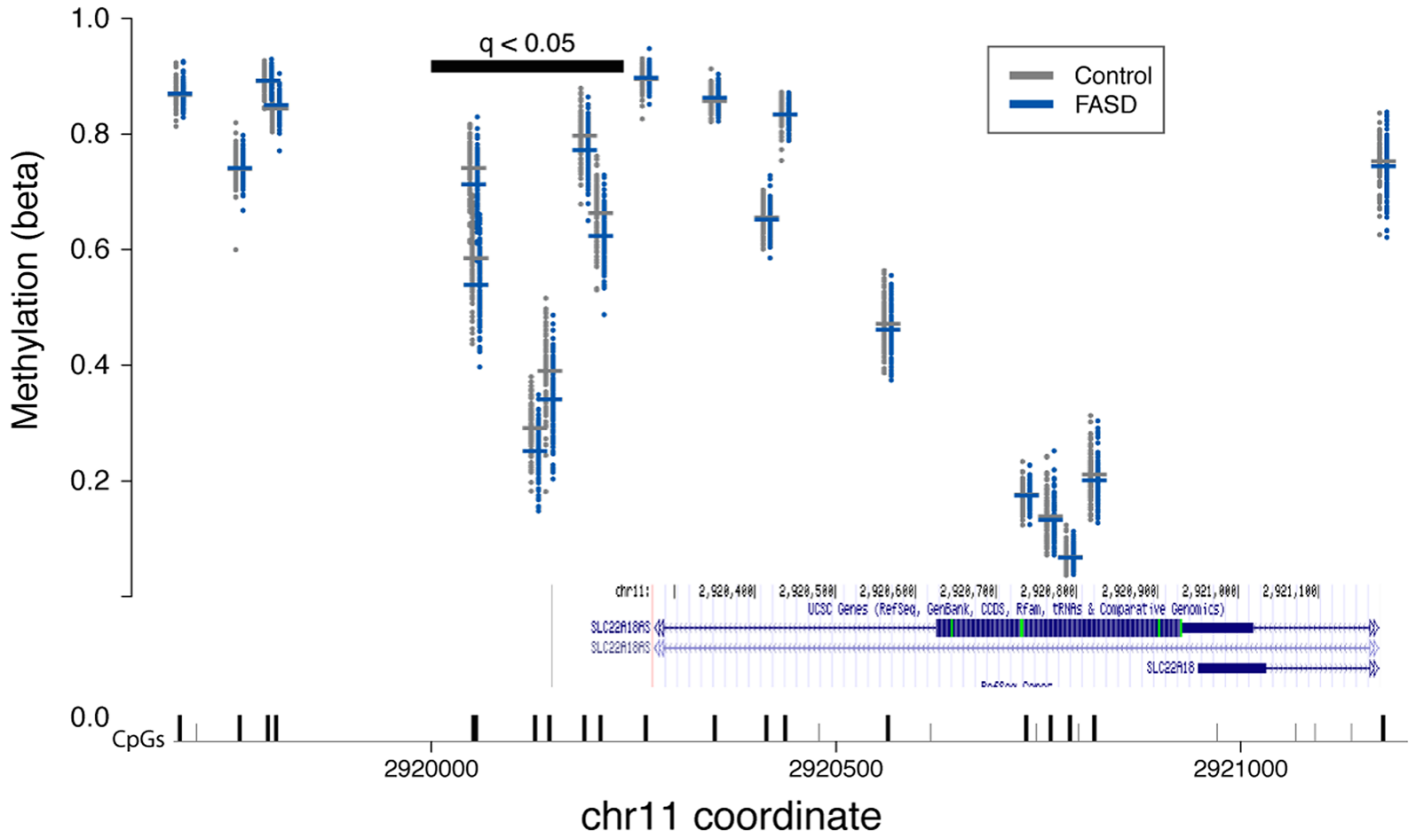
Several CpGs associated with SLC22A18 displayed down-methylation in FASD cases. The covariate-adjusted DNA methylation levels for control (*gray*) and FASD (*blue*) samples are shown for SLC22A18AS (*top*), with the gene structure aligned (*bottom*). Exons are represented by *blocks*, and transcriptional direction is indicated by *arrows*. All CpG sites are noted, those present on the 450K array are *black* while CpGs not present are *gray*. The six significantly differentially methylated probes located in the *SLC22A18* promoter region are indicated with the *horizontal black bar* [FDR-adjusted *p*-value (*q*) <0.05]

Next, the association of DM loci with different genes was assessed, with particular regards to whether some of these harbored more than one CpG differentially methylated between FASD and controls. Using genome location annotations from UCSC, the DM sites were mapped to 403 different genes. Of these, 190 were down-methylated, 208 were up-methylated, and five displayed inconsistent differences between FASD cases and controls, containing both up- and down-methylated sites, which were likely due to different genomic locations within the genes (Additional file 3: Table S2). The Phenocarta resource for gene–disease associations has previously curated a list of susceptibility genes for FASD, identifying 123 potential candidates from both human and animal studies of PAE [50]. However, DNA methylation analysis of the 115 FASD candidate genes assayed on the 450K array did not reveal significant alterations in FASD cases. Nonetheless, twelve genes contained three or more DM loci, including several genes previously involved in studies of alcohol exposure and dependence, but not present in the Phenocarta list, such as *SLC6A3* and *DRD4* (Table 3) [51–54]. This short list of DM genes also showed a slight but statistically significant enrichment for imprinted genes. The geneimprint Web site (www.geneimprint.com) currently lists 96 human genes as imprinted, 80 of which were assayed on the 450K array. Of these, five were differentially methylated in FASD cases versus controls (*ATP10A*, *CPA4*, *H19*, *KCNQ1OT1*, *SLC22A18*), with 12 out of 15 DM CpGs showing lower methylation levels in the FASD group, which resulted in a strong enrichment for imprinted probes in the list of differentially methylated probes (Fisher’s exact test; *p* = 1.8E−04). In particular, the six CpGs located within the *SLC22A18* promoter were clustered together, showing a similar pattern between FASD cases and controls, suggesting a robust regional effect of PAE on this gene’s DNA methylation profile (Fig. 4). Furthermore, 15 of the 658 DM sites were located within protocadherin genes, including six in the *PCDHB* cluster, six in the *PCDHGA* cluster, two in the *PCDHA* cluster, and one in *PCDH9*. Given the presence of multiple DM CpGs within these genes, these results provide support for imprinted genes and protocadherin clusters as strong candidates for the effects of PAE on the epigenome.

**Table 3:** Genes containing three or more differentially methylated probes

Gene	# of probes	Direction of change	Previous reports (PMID)
PCDHB gene cluster	6	Up	–
PCDHGA gene cluster	6	Up	–
SLC22A18	6	Down	20009564
H19	5	Down	21382472 19519716 19279321 20009564 23580197
HLA-DPB1	5	Up	–
DES	4	Down	–
FAM59B (GAREML)	4	Down	–
SLC38A2	4	Down	–
CAPN10	3	Up	–
DRD4	3	Down	20009564^a^
RASSF4	3	Inconsistent	–
SLC6A3	3	Up	18504048

^a^Previous reports describe change in opposite direction

### Association of FASD DM loci with neurodevelopmental processes and disorders

**Fig. 5:**
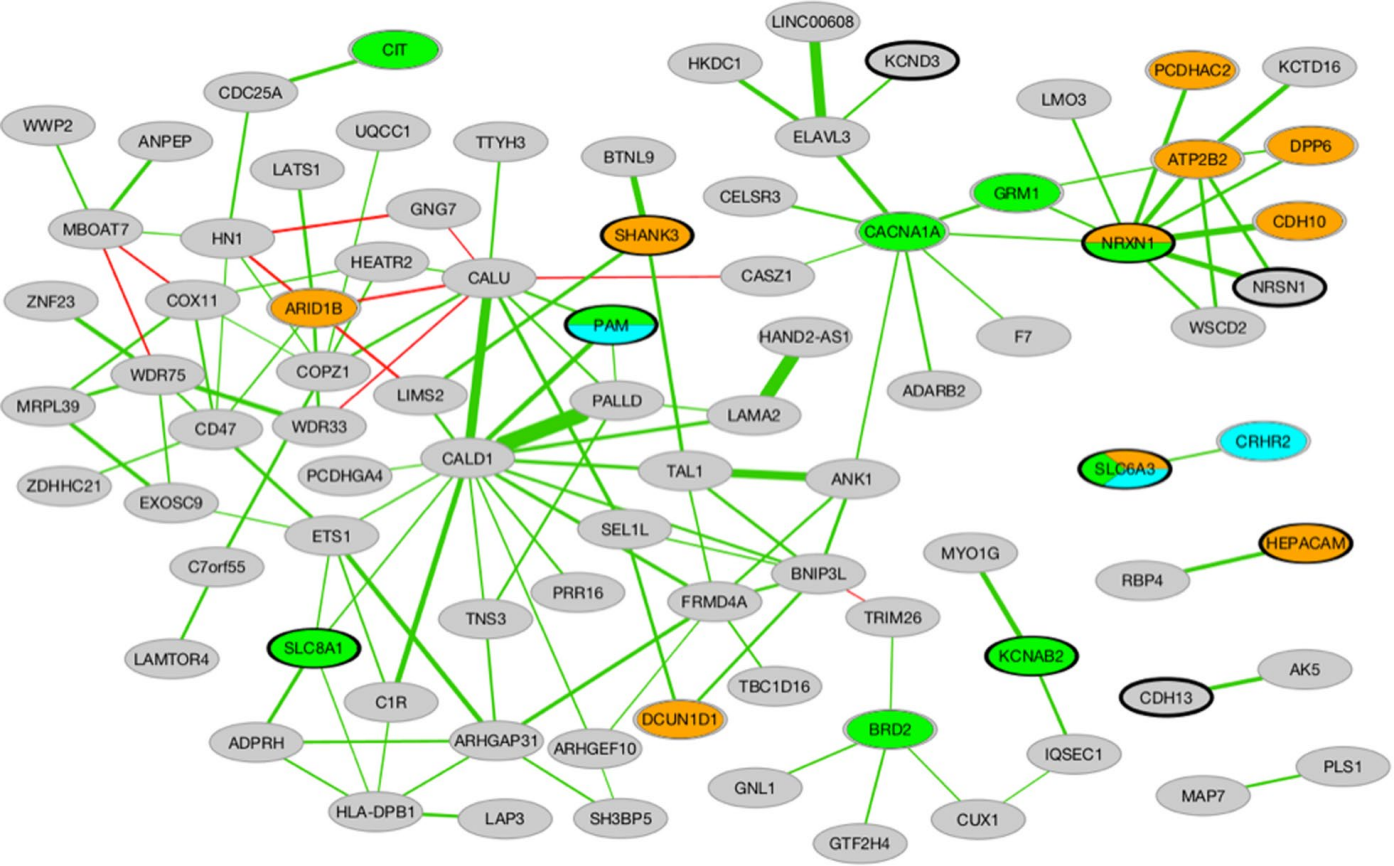
FASD up-methylated genes co-expression network. *Nodes* represent the up-methylated genes, while edges represent their co-expression link. Nodes colored in *orange*, *green*, and *cyan* are genes associated with autism spectrum disorder, epilepsy, and anxiety, respectively. The *edge width* represents the number of experiments in which the co-expression link was identified. The *green edges* show positive correlations, while the *red edges* are negative correlations.

In order to identify broad biological processes associated with altered DNA methylation patterns in FASD children, gene function enrichment analysis was performed on the dataset. As no significant results were obtained from the entire list of DM genes following multiple-test correction, the analysis was performed separately on both the up- and down-methylated gene lists. Given that the up-methylated gene list included several members of the protocadherin beta (*PCDHB*) and gamma A (*PCDHGA*) clusters, which are not differentiated by gene function annotations, a single gene from each cluster was conserved for the analysis to avoid any redundancy that may skew the results. As such, only 199 up-methylated genes and 190 down-methylated genes were analyzed for functional annotations using the overrepresentation analysis (ORA) tool in ErmineJ [55]. While no significant results were obtained using the gene ontology (GO) annotation with the list of down-methylated genes, the up-methylated gene list showed enrichment for genes associated with neurodevelopmental processes (Table 4), such as neuron parts (20 genes; FDR = 0.051) and projections (19 genes; FDR = 0.082) [50, 56]. Furthermore, using the Phenocarta annotation for associations with diseases, the list of up-methylated genes was enriched for several neurodevelopmental disorders (Table 5), including “epilepsy syndrome” (15 genes; FDR = 0.081), “autistic disorder” (12 genes; FDR = 0.092), and “anxiety disorder” (eight genes; FDR = 0.071) [50, 56]. Of note, the up-methylated genes were also marginally enriched for genes associated with substance-related disorder (15 genes; FDR = 0.192). To further examine the regulatory circuitry associated with FASD DM genes, a co-expression analysis of the up-methylated genes across 282 human expression microarray experiments, spanning multiple tissues and experimental conditions, was performed using the Gemma web tools [57]. Of the up-methylated genes, 86 could be included in the co-expression network (Fig. 5). The most strongly co-expressed pair was caldesmon 1 (*CALD1*)-Palladin (*PALLD*), which are both cytoskeleton-associated proteins [58]. In addition, a small cluster of the network showed co-expression of several genes (*NRXN1*, *CACNA1A*, *CDH10*, and others) associated with autism and/or epilepsy. Taken together, these findings suggest that altered DNA methylation patterns may potentially relate to the neurobiological deficits of children with FASD.

**Table 4:** Gene ontology function enrichment in genes up-methylated in FASD

GO name	GO ID	* p*-value	FDR	Genes
Neuron part	GO:0097458	1.38E−05	0.051	ATP2B2, CDH13, GABRB1, HEPACAM, KCNAB2, KCND3, KCTD16, NFASC, NMU, NRSN1, NRXN1, P2RX7, PAM, ROBO3, SHANK1, SHANK3, SLC6A1, SLC6A3, SLC8A1, TIAM2, UCN3
Vocalization behavior	GO:0071625	1.18E−05	0.066	NRXN1, SHANK1, SHANK3
Neuron projection	GO:0043005	7.31E−06	0.082	CDH13, GABRB1, HEPACAM, KCNAB2, KCND3, NFASC, NMU, NRSN1, NRXN1, P2RX7, PAM, ROBO3, SHANK1, SHANK3, SLC6A1, SLC6A3, SLC8A1, TIAM2, UCN3

**Table 5:** Disease association enrichment in genes up-methylated in FASD

Disease name	Disease ID	* p*-value	FDR	Genes
Anxiety disorder	DOID_2030	1.44E−04	0.071	CRHR2, CYP3A4, GRM8, NOS1AP, P2RX7, PAM, SHANK1, SLC6A3
Pervasive developmental disorder	DOID_0060040	1.15E−04	0.076	AGAP1, ARID1B, ATP2B2, ATP10A, CDH10, DCUN1D1, DPP6, ESRRB, GABRB1, GRM8, HEPACAM, NOS1AP, NRXN1, PCDHAC2, ROBO3, SDK1, SHANK1, SHANK3, SLC6A3, ST8SIA2
Epilepsy syndrome	DOID_1826	2.07E−04	0.081	BRD2, CACNA1A, CCR3, CIT, GJD2, GRM1, GRM8, KCNAB2, NRXN1, NTNG2, P2RX7, PAM, SLC6A1, SLC6A3, SLC8A1
Autistic disorder	DOID_12849	4.70E−05	0.092	AGAP1, ATP10A, CDH10, GABRB1, GRM8, HEPACAM, NOS1AP, NRXN1, ROBO3, SHANK1, SHANK3, ST8SIA2
Autism spectrum disorder	DOID_0060041	1.01E−04	0.099	AGAP1, ARID1B, ATP2B2, ATP10A, CDH10, DCUN1D1, DPP6, ESRRB, GABRB1, GRM8, HEPACAM, NOS1AP, NRXN1, PCDHAC2, ROBO3, SDK1, SHANK1, SHANK3, SLC6A3, ST8SIA2
Substance-related disorder	DOID_303	6.85E−04	0.192	ADARB2, ANPEP, CACNA1A, CDH13, CRHR2, FRMD4A, GRM8, KCND3, KISS1R, NMU, NRXN1, SLC6A1, SLC6A3, TIAM2, TRPM4

### Differentially methylated regions were identified between the FASD group and controls

**Fig. 6:**
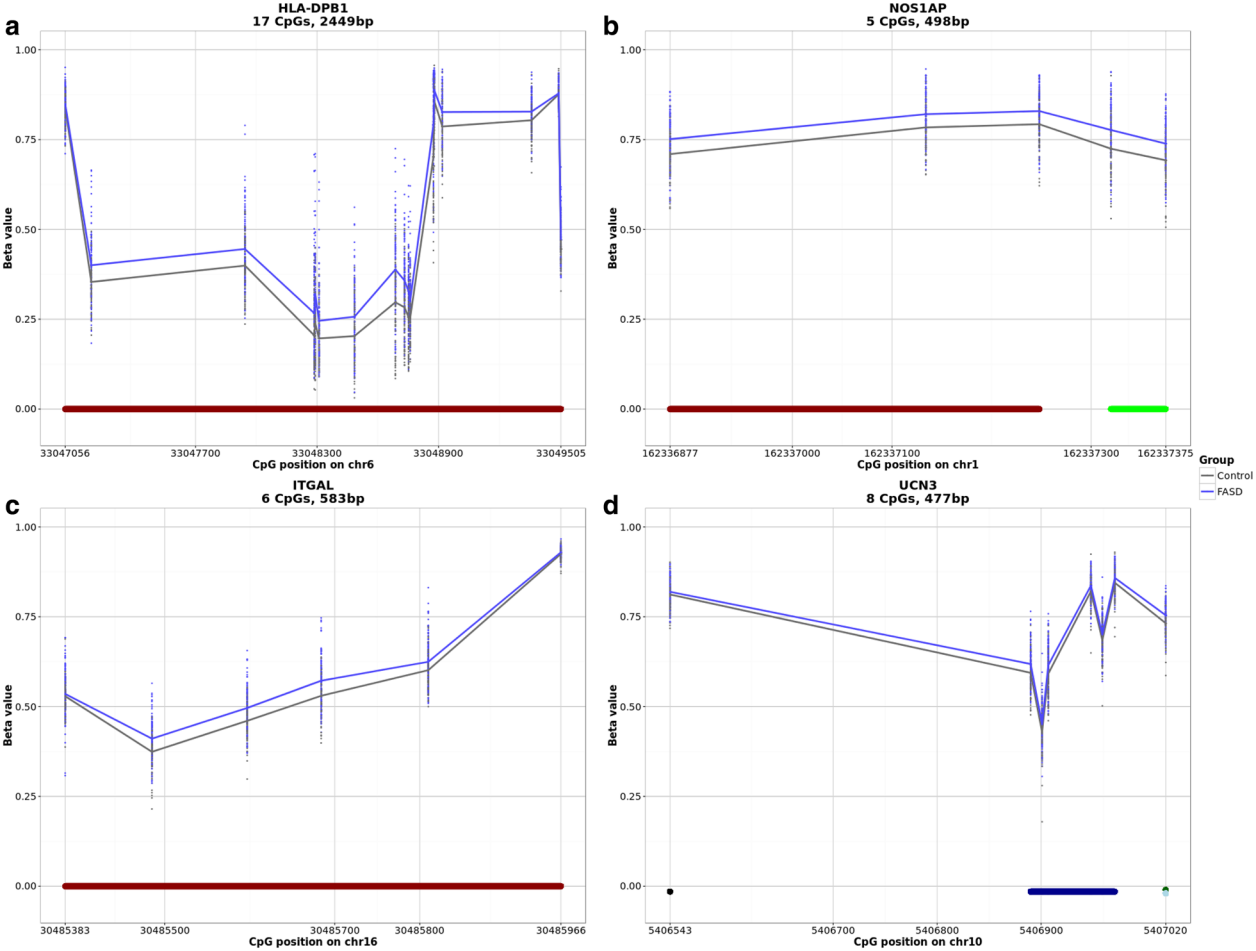
Differentially methylated regions associated with FASD. Percent methylation values adjusted for covariates were plotted across four statistically significant differentially methylated regions (DMRs) between FASD (*blue*) and controls (*gray*) identified by *DMRcate*. **a** The *HLA*-*DPB1* DMR spanned 2449 bp of the gene body (*red bar*) and contained 17 CpGs from the 450K array. **b** The *NOS1AP* DMR contained five CpGs over 498 bp and was located within the body and 3′ UTR (*green bar*) of the gene. **c** The 477 bp *UCN3* DMR contained eight CpGs. One was located within the 5′ UTR (*dark green dot*) and 1st exon (*light blue dot*), while the remainder were located upstream of the gene’s transcriptional start site (TSS), one CpG falling within 1500 bp (*black dot*) of TSS and six located within 200 bp of the TSS (*blue bar*). **d** The *ITGAL* gene contained six unique DMRs over 583 bp of the gene body (*red bar*)

To complement the site-specific analysis of differential DNA methylation, which identified several genes with multiple DM CpGs, we next attempted to identify broader patterns of differential DNA methylation using an unbiased approach. Specifically, the identification of region-specific clusters of DM CpGs between children with FASD and controls was performed using *DMRcate*, an established method that uses a Gaussian kernel smoother to identify regions of differential DNA methylation [59]. In the full dataset, 3005 differentially methylated regions (DMRs) containing two or more CpGs were identified at an FDR <0.05, while in the more homogeneous subset of samples, 289 statistically significant DMRs were identified between groups. Using the same approach to correct for the confounding effects of ethnicity as described in the site-specific analysis, 101 DMRs unbiased by ethnicity were uncovered between individuals with FASD and controls (Additional file 4: Table S3). On average, these spanned 471 nucleotides, with lower and upper limits of 31 and 2450 bp, respectively. DMRs each contained between 2 and 20 CpGs assayed on the 450K array, for a total of 504 unique sites, 75 of which were also identified in the first differential methylation analysis. Of these, 74 overlapped with 95 different genes, and 27 were located in intergenic regions. Of those associated with genes, 25 overlapped with promoter regions (within 1500 bp of the transcriptional start site), 23 with the 5′ UTR, 16 with the first exon, 49 with the gene body, and six with the 3′ UTR, as annotated from the hg19 genome assembly. Moreover, 15 of the top DMRs associated with one or more genes overlapped with those containing multiple DM CpGs in the previous analysis, including *SLC22A18*, *SLC38A2*, *HLA*-*DBP1*, and *NOS1AP* (Table 6; Fig. 6a, b). These showed the same direction of change across the entire DMR, consistent with the individual CpG differential methylation analysis and verification by pyrosequencing, in the case of *NOS1AP*. Moreover, two DMRs were identified within the protocadherin genes, with eight CpGs spanning the *PCDHGA* and *PCDHGB* clusters and four CpGs spanning the promoter of *PCDH12*, further supporting a potential role for the protocadherin genes in FASD. Importantly, in addition to the genes overlapping with the previous DM analysis, several additional DM genes were identified through this analysis, including *UCN3* and *ITGAL*, key components of the stress and immune response, respectively (Fig. 6c, d). Taken together, these results suggested that the effects of PAE on the DNA methylation went beyond single CpG loci to affect broader chromosomal neighborhoods.

**Table 6:** Top 30 gene-annotated differentially methylated regions associated with FASD

Gene symbol(s)	DMR location	Chr	Start position	End position	# of probes	Min FDR	Mean FDR	Max beta FC^a^
HLA-DPB1	Body	6	33047056	33049505	17	2.59E−50	1.61E−06	0.087
SLC22A18, SLC22A18AS	Body, TSS1500, TSS200, 5′ UTR	11	2919689	2921176	20	1.21E−29	1.46E−05	−0.049
PPP1R2P1	Body	6	32846924	32847845	18	1.81E−20	9.39E−10	0.026
SLC38A2	TSS1500	12	46767132	46768016	8	1.98E−16	9.78E−09	−0.039
HKR1	TSS1500, TSS200, 1st exon, 5′ UTR	19	37825307	37825679	7	7.51E−16	9.51E−16	0.022
WDR52	5′ UTR, 1st exon, TSS200, TSS1500	3	113160071	113160821	10	1.34E−14	6.02E−13	−0.037
C3orf24	5′ UTR, 1st exon, TSS200, TSS1500	3	10149466	10150487	11	4.41E−13	1.88E−11	0.034
NOS1AP	Body, 3′ UTR	1	162336877	162337375	5	4.69E−13	8.79E−13	0.039
KCNAB2	5′ UTR	1	6093770	6094993	6	9.78E−13	2.86E−07	0.026
F7	TSS1500, TSS200, Body	13	113759771	113760286	6	1.55E−10	1.96E−10	0.029
IFT140, TMEM204	Body	16	1598866	1599150	4	1.81E−10	4.34E−10	−0.036
RGL3	Body	19	11517079	11517436	4	3.06E−10	5.34E−10	0.036
STRA6	5′ UTR, 1st exon, TSS200, TSS1500	15	74494781	74496040	12	4.80E−10	1.06E−04	0.035
TXNRD1, EID3	5′ UTR, Body, TSS1500, TSS200, 1st exon	12	104697193	104697983	11	5.49E−10	3.98E−08	0.024
RNMTL1	Body, 3′ UTR	17	695156	695661	3	5.77E−10	3.23E−09	−0.026
C22orf42	Body, TSS200	22	32554848	32555310	5	7.95E−10	7.91E−09	0.022
RADIL	Body	7	4869981	4870162	3	2.40E−09	2.48E−09	0.026
ITGAL	Body	16	30485383	30485966	6	7.18E−09	5.13E−08	0.022
ZNF710	5′ UTR	15	90547692	90548043	3	4.18E−08	5.44E−07	−0.023
PCDHA7, PCDHAC2, PCDHA12, PCDHA6, PCDHA10, PCDHA4, PCDHA11, PCDHA8, PCDHA1, PCDHA2, PCDHA9, PCDHA13, PCDHA5, PCDHAC1, PCDHA3	Body, TSS1500	5	140344290	140344745	4	4.73E−08	1.20E−07	0.019
MAL2	TSS200, 1st exon, body	8	120220410	120221797	8	1.26E−07	2.35E−03	−0.022
UCN3	TSS1500, TSS200, 1st exon, 5′ UTR	10	5406543	5407020	8	1.32E−07	3.03E−07	0.016
HKDC1	TSS1500, 5′ UTR, 1st exon	10	70979777	70980067	4	1.37E−07	1.40E−07	0.023
ARHGEF19	Body	1	16533422	16534579	8	1.88E−07	1.11E−04	−0.035
LOC154822	Body	7	158815555	158816392	3	2.36E−07	1.90E−05	−0.043
NDST4	1st exon, 5′ UTR, TSS200, TSS1500	4	116034871	116035232	4	5.96E−07	6.45E−07	0.031
SNED1	Body	2	242009513	242009588	2	6.41E−07	6.48E−07	0.040
PRKDC	Body	8	48739161	48739256	2	7.94E−07	8.04E−07	−0.045
CASZ1	5′ UTR	1	10847541	10847594	2	2.92E−06	2.92E−06	0.025
HEATR2	Body	7	807596	809109	9	3.11E−06	3.69E−04	0.036

^a^Max fold changes (FC) represented in percent methylation change (beta) in DNA methylation levels of FASD compared to control

## Discussion

This study aimed to assess the effects of PAE on genome-wide DNA methylation patterns and identify an epigenetic signature of FASD, using a large cohort of human subjects. Significant changes to the DNA methylation profiles in BECs of children with FASD compared to age- and sex-matched typically developing controls were identified, with 658 CpGs displaying significantly altered DNA methylation levels, of which 41 had a >5 % methylation change. Moreover, 101 DMRs containing two or more sequential DM CpGs were identified throughout the genome, spanning 95 different genes, overlapping with several from the initial differential methylation analysis at single CpG level. The majority of DM genes were highly expressed in postmortem brain samples from the Allen Brain Institute. Moreover, BEC and independent cortical samples showed relatively high concordance of DNA methylation levels. As discussed in more detail below, several lines of evidence converge to support the validity of our data. First, a number of DM sites and regions were identified within genes and pathways previously associated with PAE. Second, novel DM sites and regions tended to be involved in pathways implicated in functional deficits of FASD. Third, broader patterns related to altered neurodevelopmental disorders were identified in sets and networks of genes associated with FASD in our study.

Differential DNA methylation analysis in our case–control study comparing children with FASD to children with typical development replicated several associations from previous studies of PAE. One of the most striking similarities is the altered DNA methylation patterns observed in imprinted genes. Several studies have demonstrated the effect of PAE on the *H19* imprinted gene in both mice and humans [31, 60, 61]. A genome-wide DNA methylation study in mouse embryos exposed to ethanol also identified significant changes within several imprinted genes including both *H19* and *SLC22A18* [38]. Results from our study further confirmed these findings, as five down-methylated probes in *H19* and six in *SLC22A18* were altered in the FASD cohort, with the latter being identified as a broader DMR as well. Given that imprinting plays a key role in the regulation of normal growth and development, its alteration by alcohol exposure could be a factor in the neurodevelopmental defects observed in children with FASD [62]. Furthermore, the only other study of genome-wide DNA methylation patterns in individuals with FASD also identified several DM protocadherin genes within the alpha, beta, and gamma clusters, though only one CpG overlapped with the results presented here [40]. The differences in specific CpGs within these gene clusters between the two studies might be due to the much larger sample size of our study, as well as our use of multiple-test correction to mitigate spurious patterns of differential DNA methylation associated with the FASD group. However, we note that the single CpG site from our study that overlapped with the previous findings (cg21117330) was located in *PCDHGA8* and displayed the same direction of change between FASD cases and controls and thus might represent a robust and reproducible effect of PAE.

In addition to genes previously identified in studies of PAE, DNA methylation changes were also uncovered in a number of additional genes with functional relevance to the deficits observed in FASD. More specifically, analysis of DM probes and regions identified altered DNA methylation patterns within genes related to the immune response, such as *HLA*-*DPB1*, a HLA class II histocompatibility antigen, and *ITGAL* (or *CD11A*), the integrin alpha L chain. Given that children with FASD often present with numerous deficits in immune function, epigenetic alterations of these genes might reflect functionally relevant underlying biology [63]. A DMR between FASD cases and controls was also identified in *UCN3*, an antagonist of the CRF type 2 receptor, which plays a key role in the stress response. As this gene acts downstream of stress signaling pathways, this alteration might be linked to altered basal levels of corticosterone found in individuals with FASD [1, 64]. Finally, two members of the dopaminergic system, *SLC6A3* and *DRD4*, each contained three differentially methylated CpGs in FASD cases compared to controls. Both of these genes have also been proposed as modifiers and/or risk factors in alcohol abuse disorders and attention deficit disorder, and thus might potentially play a role in the deficits of attention and executive function in children with FASD [53, 54].

Moving beyond alterations in specific genes related to PAE, broader associations to neurodevelopmental processes and disorders were identified in genes containing differentially methylated CpGs. In particular, the gene co-expression network contained a small sub-network of genes associated with autism and/or epilepsy, and up-methylated genes in FASD cases were enriched for functions related to neurodevelopmental disorders. These results could reflect the pleiotropy of these genes, or perhaps their involvement in developmental functions dysregulated in neurodevelopmental disorders with partially overlapping phenotypes. As many of these genes were also functionally enriched for neuron parts and projections, they could influence processes necessary for typical brain development and partially underlie some deficits observed in children with FASD and other neurodevelopmental disorders.

Comparing epigenetic patterns associated with FASD and autism presented an interesting conundrum. While we identified a small sub-network of genes associated with autism and/or epilepsy in our analysis of the FASD-related gene co-expression network, this relationship did not extend to the level of individual CpGs. Comparing the 14 DM genes from BECs recently reported to be associated with autism spectrum disorder, we did not find any overlap with the DM loci identified in our study of FASD children [46]. The differences between the gene lists may reflect the different origins and phenotypes between the conditions, or that the effects of PAE are more easily identifiable in peripheral tissue than those of autism, or simply false positives and/or false negatives. Regardless, these results imply that at the single CpG level, genes showing differences in DNA methylation between FASD cases and controls are reflective of FASD-specific alterations, rather than broad neurodevelopmental functions.

Although it is tempting to speculate that our collective results may be partially related to the functional deficits observed in FASD, it is important to consider that the DNA methylation patterns were derived from BECs. We feel that this concern is partially mitigated by our finding of the majority of DM genes in BECs being consistently expressed in the brain and by the DNA methylation patterns in neural tissue displaying high correlation with those in BEC. Moreover, it has been noted by others that BECs might be a good surrogate tissue for human DNA methylation studies, as both buccal and brain cells are derived from the ectoderm [65]. Lastly, while our study did not measure DNA methylation in additional tissues, evidence from animal models is emerging to support lasting alterations to both epigenetic and gene expression patterns in neural tissue following PAE [39, 66–68]. Nevertheless, our results must be interpreted with caution in the context of neurodevelopment, as additional studies in postmortem samples from humans are required to rigorously assess the concordance of epigenetic changes associated with FASD between peripheral and central tissues.

A further challenge in the interpretation of alterations to DNA methylation patterns in FASD cases versus controls lies in the small effect sizes of environmental exposures on the epigenome. Although the small DNA methylation changes observed here are consistent with genome-wide DNA methylation studies in other neurodevelopmental and psychiatric disorders, it is unclear whether such small changes can have a strong effect on cellular functions [45, 46, 69]. However, slight changes accumulating in several genes involved in similar processes could combine to have strong effects on biological processes. For instance, as many of the up-methylated genes were co-expressed, small alterations to multiple members of this network could potentially affect the biological functions they regulate.

While our data are very consistent with published work in human epigenome-wide association studies, it is of course possible that the relatively small changes to DNA methylation levels reflect biological biases or even technical noise [69]. These could originate from a variety of sources, which we attempted to address to the best of our abilities. For example, although differences in cell-type composition can play an important role in driving DNA methylation variation, no contamination of the BEC from the present study with white blood cells was identified, suggesting that differences in cell-type composition likely did not affect the observed alterations to DNA methylation patterns in the FASD group. In addition to differences in cell types, differing postnatal environments between groups might also influence the observed DNA methylation patterns, skewing the results to represent possibly confounding variables other than PAE, such as diet, SES, and postnatal alcohol exposure. However, the majority of children in the FASD group were living in foster or adoptive homes, rather than the biological family, which hopefully would reduce differences in the rates of alcohol use or food security between groups. By contrast, SES scores were slightly confounded between groups, although this effect was partially mitigated by the focus on the more ethnically homogeneous subgroup, which showed less skewing toward low SES in the FASD cases. Finally, we feel that potential technical issues were reduced through the use of strict quality control and statistical procedures to eliminate unwanted variation in the data. As such, the technical validity of our approach was supported by the verification of five DM loci by bisulfite pyrosequencing, the gold standard for targeted DNA methylation analysis.

We note that although most biological and technical issues were addressed by our study design and methods, a particular caveat in the identification of DM loci was manifested by the imbalance in ethnicity across FASD cases and control groups. Other studies have included ethnicity as a covariate during linear modeling to correct for its effects, but no significant DM probes were identified using this approach in our study, as FASD status was confounded with ethnic background (Additional file 1: Supplemental methods). Given that self-reports do not always accurately assess ethnicity, SNP genotyping data were used to objectively assign participants to different ethnic groups, based on HapMap samples of known ethnicity. This analysis resulted in the identification of a more homogeneous subgroup of samples, which was used as a comparative control to filter out the influences of ethnicity and related effects, such as SES and cultural confounders, on differential DNA methylation within FASD cases. In turn, this strategy facilitated the removal of ethnically biased probes from the original DM loci, resulting in the successful identification DM CpG sites specific to children with FASD and not confounded for ethnicity. Given the prevalence of ethnically diverse populations in large-scale studies of DNA methylation, this unique approach driven by genetic stratification of subgroups might prove a useful way of dealing with the effects of ethnicity in case–control studies beyond the one presented here.

## Summary and conclusions

Despite the recognition of FAS over 40 years ago, PAE remains a leading cause of developmental disability in the developed world. While several animal studies have investigated the role of epigenetic mechanisms in the context of PAE, most human studies have been limited to alcohol consumption and dependence in adults, or a small cohort of children with FASD [40, 52, 70, 71]. As such, this study is the single largest investigation of genome-wide DNA methylation patterns in children with FASD. While one of the greatest challenges with this large cohort was the ethnicity imbalance between the FASD and control groups, ethnic background correction reduced this confound and allowed the reliable identification of 658 DM CpG sites specific to children with FASD. Although the effect size of changes was small in some cases, 41 sites displayed a >5 % change in DNA methylation, which is consistent with previous studies and may reflect the subtle effects of PAE on the epigenome. We also identified 101 DMRs containing two or more DM CpGs, located within 95 different genes and spanning promoter regions, gene bodies, and both 3′ and 5′ UTRs. While these data were collected from BEC, rather than neural tissue, the vast majority of DM genes were highly expressed in the brain, suggesting a potential concordance between peripheral and central tissues. These alterations occurred in several genes previously implicated with PAE and altered neurodevelopment, and displayed functional enrichments for neural process and neurodevelopmental disorders. Although it will be essential to validate these changes in separate cohorts from a different population, these findings provide initial insight into the molecular mechanisms underlying the effects of PAE on children and present a potential role for role for DNA methylation in the etiology of FASD.

## Methods

### Participants and samples

Ethics for this project were reviewed and approved by the “Children’s and Women’s Research Ethics Board—Clinical” (H10-01149). Children with FASD and age- and sex-matched typically developing children were recruited from multiple FASD diagnostic clinics across Canada, where saliva samples and BECs were collected for genotyping and DNA methylation analysis, respectively [41]. All experimental procedures were reviewed and approved by the Health Research Ethics Boards at Queen’s University, University of Alberta, Children’s Hospital of Eastern Ontario, University of Manitoba, and the University of British Columbia. Written informed consent was obtained from a parent or legal guardian, and assent was obtained from each child before study participation. The majority of clinics used previously described guidelines for the diagnosis of FASD [72]. Briefly, samples were collected from 112 FASD and 102 age- and sex-matched control children aged between 5 and 18 (Table 1). Saliva samples were collected using the Oragene DNA kit (DNA Genotek Inc., Ontario, Canada) according to the manufacturer’s instructions. BECs were collected using the Isohelix buccal swabs and Dri-Capsule (Cell Projects Ltd., Kent, UK). To collect buccal cells, the swab was inserted into the participants’ mouth and rubbed firmly against the inside of the left cheek for 1 min. The swab was then placed into a sterile tube with a Dri-Capsule and the tube sealed. An identical procedure was followed for the right cheek. Participants did not have any dental work performed 48 h prior to collection, and no food was consumed <60 min prior to collection to avoid contamination.

### DNA methylation 450K assay

DNA was extracted from buccal swabs using the Isohelix DNA isolation kit (Cell Projects, Kent, UK). Seven hundred and fifty nanograms of genomic DNA was subjected to bisulfite conversion using the Zymo EZ DNA Methylation Kit (Zymo Research, Irvine, CA, USA), which converts DNA methylation information into sequence base differences by deaminating unmethylated cytosines to uracil while leaving methylated cytosines unchanged. One hundred and sixty nanograms of converted DNA was applied to the HumanMethylation450 BeadChip array from Illumina (450K array), which enables the simultaneous quantitative measurements of 485,512 CpG sites across the human genome, following the manufacturer’s instructions. Chips were scanned on an Illumina HiScan, with the 214 samples run in two batches and each containing an equal number of FASD and control samples, randomly distributed across the chips. Two pairs of technical replicates were included and showed a Pearson correlation coefficient *r* > 0.996 in both cases, highlighting the technology’s reproducibility.

### DNA methylation data quality control and normalization

The raw DNA methylation data were subjected to a set of rigorous quality controls, first of the samples and then of the probes. Of the 214 initial samples, eight were removed from the final dataset due to various quality and concordance issues. Of these, five were removed based on poor quality data, which was identified through skewed internal controls and/or ≥5 % of probes with a detection *p*-value >0.05. One sample was removed due to a gross chromosomal abnormality identified in the genotyping and DNA methylation data. The genotypes of the samples, based on the 65 SNP probes contained on the 450K array, were compared to the genotypes from the SNP arrays. The genotypes were highly correlated for all samples (Pearson correlation coefficient *r* > 0.9), except one, which was excluded from further analyses. Finally, as a pair of monozygotic twins was present in the control group, only one of their samples was chosen at random and retained in the analysis to remove any genetic bias. Next, probes were removed from the dataset according to the following criteria: (1) probes on X and Y chromosomes (*N* = 11,648); (2) SNP probes (*N* = 65); (3) probes with beadcount <3 in 5 % of samples (*N* = 3029); (4) probes with 1 % of samples with a detection *p*-value >0.05 (*N* = 10,163); or (5) probes with a polymorphic CpG and nonspecific probes as defined by the Price annotation (*N* = 20,869 SNP-CpG and 41,937 nonspecific probes; [42]). A final filtering step was performed to set the methylation values to NA for any remaining probe–sample pair where beadcount <3 or detection *p*-value >0.05. Data normalization was performed using the beta-mixture quantile normalization method on the final dataset, composed of 206 samples (110 FASD and 96 controls) and 404,030 probes [73]. All analyses were performed using *M* values, which represent the log 2 ratio of methylated/unmethylated, where negative values indicate <50 % methylation and positive values indicate more than 50 % methylation [74]. Percent methylation changes (beta-values) were used in graphical representations of the data and indicate the percentage of methylation calculated by methylated/(methylated + unmethylated), ranging from 0 (fully unmethylated) to 1 (fully methylated).

### Differential methylation analysis

Given that DNA methylation changes are typically small and that unknown sources of variation, including cellular heterogeneity, may influence the data, SVA was performed to identify SVs representative of unwanted heterogeneity using the SVA package in R [75]. Using DNA methylation data from all 206 samples, SVA identified 15 SVs not associated with clinical status (FASD vs control), which, as expected, were only partially correlated with known covariates (Additional file 1: Supplemental methods, Figure S2). Linear regression analysis was performed on the dataset with the *limma* package in R, utilizing a model that included clinical status and all identified SVs as covariates [76]. Statistically significant differences between groups were required to show a false discovery rate (FDR) <0.05 following multiple-test correction by the Benjamini–Hochberg method [77]. Further evaluation of potential biological significance was assessed by mean percent DNA methylation differences between FASD and controls.

### Analysis of effects due to familial and diagnosis status

As the cohort included several sets of siblings and cousins, a sensitivity analysis was performed to identify potential family effects in the dataset. However, little effect of familial origin was observed, indicating that the presence of families in the cohort did not significantly impact the study’s results or require statistical correction (Additional file 1: Supplemental methods). Furthermore, this cohort also included children with PAE that were not formally diagnosed with FASD (27 children). As such, additional differential DNA methylation analyses were performed on the two individual subgroups of FASD cases compared to controls (Additional file 1: Supplemental methods). However, as these did not reveal any significant differences between diagnosed FASD cases and PAE children, the PAE cases were included in the FASD group for all analyses.

### Genotyping

Genomic DNA was extracted from saliva samples following standard procedures. Briefly, 161 DNA samples were genotyped for 2,443,177 markers using the Infinium HumanOmni2.5-Quad v1.0 BeadChip (Illumina Inc., San Diego, CA, USA) and 54 samples were genotyped for 2,379,855 markers using the Infinium HumanOmni2.5-8 v1.0 BeadChip (Illumina Inc., San Diego, CA, USA) according to the manufacturer’s protocol. For both microarrays, 200 ng of DNA (4 μL at 50 ng/μL) was independently amplified, labeled, and hybridized to BeadChips and then scanned with default settings using the Illumina iScan. Analysis and intra-chip normalization of resulting image files were performed using Illumina’s GenomeStudio Genotyping Module software v.2011 with default parameters. Genotype calls were generated using the Illumina-provided genotype cluster definitions files (HumanOmni2.5-4v1_H.egt and HumanOmni2.5-8v1_C.egt generated using HapMap project DNA samples) with a Gencall cutoff of 0.15. Only the 2,368,900 common SNPs were used for analysis. pyGenClean v1.2.2 and PLINK v1.07 were used for quality control and genetic data cleanup process, respectively. SNPs with completion rate <98 %, uninformative (MAF = 0) and failed for Hardy–Weinberg equilibrium exact test (*p*-value <2.9 × 10^−8^) were removed. Samples with completion rate <95 % were excluded.

### Subsample definition

MDS was performed on the participants’ genotype data including 83 founder individuals from the Caucasian population (CEU), 186 from the Japanese and Han Chinese population (JPT-CHB), and 88 from the Yoruba population (YRI) (HapMap; [78]).

All 195 samples that had both genotyping and DNA methylation data were hierarchically clustered based on the first four principal components from the MDS analysis. One individual of African descent was excluded because of their unique ethnicity compared to the rest. All other samples clustered in two groups: Cluster 1 = 136 samples (49 FASD:87 controls) and Cluster 2 = 58 samples (53 FASD:5 control) (Additional file 1: Figure S3). Cluster 1 was selected as the more balanced subsample, in terms of both ethnicity and cases versus controls, for further analysis (see Fig. 1 for a summary of the bioinformatic analyses).

### Ethnic group adjustment

Differential DNA methylation analysis was performed as previously described on the more genetically homogenous subsample defined as “Cluster 1” in the MDS analysis above to identify difference between FASD cases and controls. SVA using this subsample identified 11 SVs that were added as covariates in linear modeling, as described for the full sample. Ethnically confounded probes were explored in more detail to ensure that the adjustment was performing as expected (Additional file 1: Supplemental methods, Figure S4). In addition, the inclusion of principal components from the MDS analysis into the regression model to correct for ethnicity was also explored. However, as ethnicity was confounded with the phenotype of interest, direct correction in the model also removed the signal of interest (Additional file 1: Supplemental methods).

### DNA methylation pyrosequencing assay

Bisulfite pyrosequencing assays were designed with PyroMark Assay Design 2.0 (Qiagen; Additional file 5: Table S4). The regions of interest were amplified by PCR using the HotstarTaq DNA polymerase kit (Qiagen) as follows: 15 min at 95 °C, 45 cycles of 95 °C for 30 s, 58 °C for 30 s, and 72 °C for 30 s, and a 5 min 72 °C final extension step. For pyrosequencing, single-stranded DNA was prepared from the PCR product with the Pyromark™ Vacuum Prep Workstation (Qiagen), and the sequencing was performed using sequencing primers on a Pyromark™ Q96 MD pyrosequencer (Qiagen). The quantitative levels of methylation for each CpG dinucleotide were calculated with Pyro Q-CpG software (Qiagen).

### Brain concordance analysis

Human brain blood DNA methylation data from a previously published cohort were used to assess concordance, which was calculated as the Spearman correlation coefficient of DNA methylation at all CpGs between healthy human blood and brain [48]. Human brain microarray data were obtained from the Allen Brain Atlas (http://human.brain-map.org/static/download), which contains normalized expression values for 58,692 probes and 896 brain regions from six individuals. Probes were ranked based on their average expression level for each brain region separately, and the mean was calculated across all brain regions. All 29,191 genes assayed (which included 389 out of our 404 differentially methylated genes) were sorted based on their highest ranked probe.

### CpG island distribution

The probes categorization into “North Shelf,” “North Shore,” “Core Island,” “South Shore,” “South Shelf,” or “Non-island” was based on the Illumina “RELATION_TO_UCSC_CPG_ISLAND” annotation. The expected counts were calculated with the 404,030 probes remaining after filtering. Statistics were calculated using multinomial goodness-of-fit Chi-square test. As a post hoc test to evaluate which category is driving the effect, additional Chi-square tests were run on each category versus the sum of all of the other categories.

### Functional enrichment analysis

The list of imprinted genes was extracted from http://www.geneimprint.com/site/genes-by-species.Homo+sapiens.imprinted-All (Additional file 6: Table S5), which includes 80 genes with at least one probe among the 404,030 probes remaining after filtering (3035 probes total). The Illumina “UCSC_REFGENE_NAME” annotation was used to map the probes to genes (479 out of 658 DM probes had such annotation and could be mapped). In the event of probes mapping to several genes, the gene with the closest transcription start site (TSS) was selected using the Price annotation [42]. The ORA tool of ermineJ (version 3.0.2) was used to identify gene function enrichment in the list of up- and down-methylated genes including the GO annotations molecular function, biological process, and cellular component [55]. The ermineJ ORA tool was set with the following parameters: max gene set size = 1000; min gene set size = 2; background genes = all genes mapping to the 404,030 probes remaining after filtering.

### Co-expression analysis

The Gemma tools and database for meta-analysis of functional genomics data were used to perform a co-expression analysis based on existing studies [57]. The methods used by Gemma have been previously described [79]. Datasets were obtained from public sources, primarily the Gene Expression Omnibus [80]. For each dataset included in the meta-analysis, the Pearson correlation matrix of gene co-expression profiles was computed. Thresholds were applied for statistical significance of correlation, and the resulting sparse co-expression networks were aggregated across datasets. The degree to which a link is replicated across studies is a measure of its reliability; a threshold was set based on a benchmark permutation-based analysis, scaled to the number of datasets aggregated. Using the Gemma online tools, a co-expression network was extracted for the 199 up-methylated genes in the master set of microarray experiments for human (282 usable experiments across multiple tissues and experimental conditions) at the stringency recommended by the software, and visualized the results in Cytoscape [81]. The resulting network shows the co-expression relationship of the genes in the input list only.

### Differentially methylated region analysis

The identification of DMRs was performed using previously established guidelines and the *DMRcate* package in R [59, 82]. Briefly, results from linear modeling with SVs were analyzed using a Gaussian kernel smoother with a bandwidth of 1000 bp and scaling factor of 2 to model all CpG sites in the genome in parallel and identify broad regions of differential DNA methylation. *p*-values were corrected for multiple testing using the BH method, and an FDR cutoff of 0.05 was used to select significant probes between the FASD and control groups. DMRs were then assigned by clustering significant CpGs located within 1000 bp windows that contained two or more CpGs. This analysis was performed on both the full dataset and the more ethnically homogeneous subset of individuals, and the final list of DMRs was obtained through the same process as previously described in the differential methylation analysis. Genomic locations for all DMRs were assigned using the Illumina hg19 annotation.

## Additional files

10.1186/s13072-016-0074-4 Supplementary methods include additional information on SVA, familial effects analysis, PAE vs FASD analyses, and further investigation of ethnicity-related effects on DNA methylation. Fig S1 displays the principal component analysis of BEC versus blood samples. Fig S2 shows the correlation of SVs with different variables. Fig S3 shows the clustering of samples based on genotype. Fig S4 shows the effect of ethnicity adjustment on significantly DM probes. Fig S5 shows the distribution of SES scores between FASD and control groups. Fig S6 shows the correlation between pyrosequencing and 450K array results. Fig S7 shows the correlation of BEC and brain DNA methylation. Fig S8 shows the distribution of DNA methylation levels. Fig S9 shows the distribution of *p*-values for PAE, FASD, and control contrasts (described in additional file 1).
10.1186/s13072-016-0074-4 Table of differentially methylated probes.
10.1186/s13072-016-0074-4 Table of genes with multiple differentially methylated probes.
10.1186/s13072-016-0074-4 Table of differentially methylated regions.
10.1186/s13072-016-0074-4 Table of pyrosequencing primers.
10.1186/s13072-016-0074-4 Table of imprinted genes in humans.

## Abbreviations

450K array: Illumina HumanMethylation450 BeadChip array
BEC: buccal epithelial cell
CpG: cytosine–guanine dinucleotide
CGI: CpG island
DM: differentially methylated
DMR: differentially methylated region
FASD: fetal alcohol spectrum disorder
FDR: false discovery rate
GO: gene ontology
MDS: multi-dimensional scaling
mQTL: methylation quantitative trait loci
ORA: overrepresentation analysis
PAE: prenatal alcohol exposure
ROC: receiver operating characteristic
SNP: single nucleotide polymorphism
SEP: socioeconomic position
SV: surrogate variable
SVA: surrogate variable analysis
TSS: transcription start site
UCSC: University of California, Santa Cruz Genome Browser
UTR: untranslated region
