# Supplementary material for: DNA methylation signature of human fetal alcohol spectrum disorder
Source: Epigenetics Chromatin. 2016 Jun 29;9:25. doi: 10.1186/s13072-016-0074-4 (PMC4926300; doi:10.1186/s13072-016-0074-4)
Supplement: Supplementary file 1 — 10.1186/s13072-016-0074-4 Supplementary methods include additional information on SVA, familial effects analysis, PAE vs FASD analyses, and further investigation of ethnicity-related effects on DNA methylation. Fig S1 displays the principal component analysis of BEC versus blood samples. Fig S2 shows the correlation of SVs with different variables. Fig S3 shows the clustering of samples based on genotype. Fig S4 shows the effect of ethnicity adjustment on significantly DM probes. Fig S5 shows the distribution of SES scores between FASD and control groups. Fig S6 shows the correlation between pyrosequencing and 450K array results. Fig S7 shows the correlation of BEC and brain DNA methylation. Fig S8 shows the distribution of DNA methylation levels. Fig S9 shows the distribution of p-values for PAE, FASD, and control contrasts (described in additional file 1). [file 13072_2016_74_MOESM1_ESM.docx]

# Supplemental methods

## Surrogate variable analysis (SVA)

The SVA with the full sample resulted in the identification of 15 surrogate variables (SVs), with the first 2 SVs representing 27 and 20% of the variance respectively and the others less than 6% each (Figure S2A). We applied linear regression to each SV using each known covariate as potential predictor. We tested 8 known covariates: Run (2 levels = dates when 450k arrays were processed), Chip (18 levels = Chip carrying the sample), location (96 levels = sample location on the array in each run), Plate_column (8 levels = column in 96-well plates), Plate_row (12 levels = row in 96-well plates), Gender (2 levels), Race (24 levels = self-declared ethnicity), and Age (continuous). For the discrete variables with more than two levels, the multiple regression model was built with dummy variables. This results in models that include different amounts of variables. To compare the different models against each other, we obtained the R squared values, adjusted for the number of variables in each model (Figure S2B). Most surrogate variables are best explained by technical covariates and less by biological covariates. However, it is clear that a lot of the variance remains unexplained by known covariates and justifies our use of the SVs in our model to better account for unknown undesirable variation.

## Sensitivity analysis for family effect

The data includes 44 sets of siblings/cousins. To investigate whether this impacts our results, we ran an additional differential methylation analysis on the full sample, using the same model that includes the clinical factor (FASD vs. Control) + all 15 SVs identified, with the addition of ‘Family’ as a random effect, using the lme4 R package. We compared the results from this analysis to the original analysis. The Spearman rank correlation is 0.9996. There are 1658 significant probes, 1601 overlap with the 1661 probes from the original analysis, 639 overlap with the 658 final probes after ethnicity adjustment (Additional file 2). The difference is minor and we conclude that the presence of families in our cohort do not impact significantly our results.

## PAE vs FASD analysis

We performed an additional differential methylation analysis after excluding 27 FASD samples with no official diagnosis (denoted PAE for Prenatal Alcohol Exposure). This analysis, including 96 controls and 83 FASDd (FASD with diagnosis), resulted in 502 significant DM probes at a FDR of 0.05, 461 of which overlap with the 1661 probes from the original analysis. The Spearman rank correlation between the results from the original analysis and the analysis without PAE is 0.7927. In addition, we ran ANOVA analyses for the 658 significant probes after ethnicity adjustment, on Control vs. FASDd, Control vs. PAE, and FASDd vs. PAE. We observed that, for most of the probes, methylation in controls is significantly different from either FASDd or PAE, while FASDd vs. PAE doesn’t lead to any significant results (Figure S9). We concluded from these results that the PAE samples are not significantly different from the FASDd samples and should be kept in the analysis as part of the broad FASD group.

## Investigating ethnically-biased probes

To investigate ethnically-biased probes (denoted “ethnic” probes below), we ran a differential methylation analysis on the FASD samples, looking for differences between samples from the 2 ethnic clusters identified in the MDS analysis (49 samples in cluster 1 vs. 53 samples in cluster 2). It is important to note that the ethnicity bias is directional – the FASD Group is confounded with the ethnic cluster 2 and this is only problematic when the direction of change is the same (e.g. higher in FASD and higher in cluster 2). We thus calculated the one-sided p values and rank for all probes in the above analysis and investigated separately the up-methylated and down-methylated “ethnic” probes. We obtained 1105 probes significantly differentially up-methylated (FDR 0.05) and 594 probes differentially down-methylated (FDR 0.05) in the ethnic cluster 2. These overlap with 5 up-methylated and 4 down-methylated probes in FASD respectively, out of the 658 significant probes after ethnicity adjustment (Additional file 2). To investigate whether the ethnicity adjustment we performed was efficient at removing “ethnic” probes, we looked at the “ethnic” ranking of our significant FASD DM probes (Figure S4A). We performed a Receiver Operating Characteristic (ROC) analysis with the ethnic probes (Figure S4B). Our approach was to question how good the ethnic ranking was at predicting the 658 FASD DM probes, a high AUC indicating that the DM probes are in the top of the “ethnic” rank and thus very highly biased. Our results show that while the “ethnic” ranking can somewhat predict our 1661 FASD DM probes (AUC = 0.819 and 0.773 for up- and down-methylated probes respectively), it is much less effective at predicting our 658 FASD DM probes after ethnicity adjustment (AUC = 0.656 and 0.594 for up- and down-methylated probes respectively). These results confirm that our ethnicity adjustment is reducing the ethnic bias as expected.

## Incorporating ethnicity as a set of covariates in the regression model

We investigated the option of incorporating the ethnicity as a covariate in the model, using the genotyping data available. There are 195 samples with both genotyping and methylation data, 103 FASD and 92 controls. We tested different models and decided to perform a linear regression analysis using Limma with a model that includes the clinical factor (FASD vs. Control) + Gender + C1 + C2 + C3 (First 3 components from the MDS analysis) + family as a random factor. The multiple-testing adjusted significance threshold was established at p = 1.2 x 10-7 (0.05/404030). No significant probes could be identified. This is expected because the FASD status is confounded with ethnicity and correcting for one will erase the small changes the can be observed in the other.

**Supplemental figures**

**Fig S1. Principal component analysis of buccal epithelial cell and blood samples.** We obtained DNA Methylation data from 541 peripheral blood leukocyte samples (GSE42861) from the Gene Expression Omnibus (GEO) public database. We performed a principal component analysis including these 541 samples together with our 206 FASD samples. The first two principal components, which typically associate with cell type, are depicted for the buccal epithelial cell (BEC) samples from the present study and blood samples obtained from the Gene Expression Omnibus (those beginning with GSM). As shown here, both tissue types cluster separately and little to no blood contamination of BECs is apparent in the dataset.

**
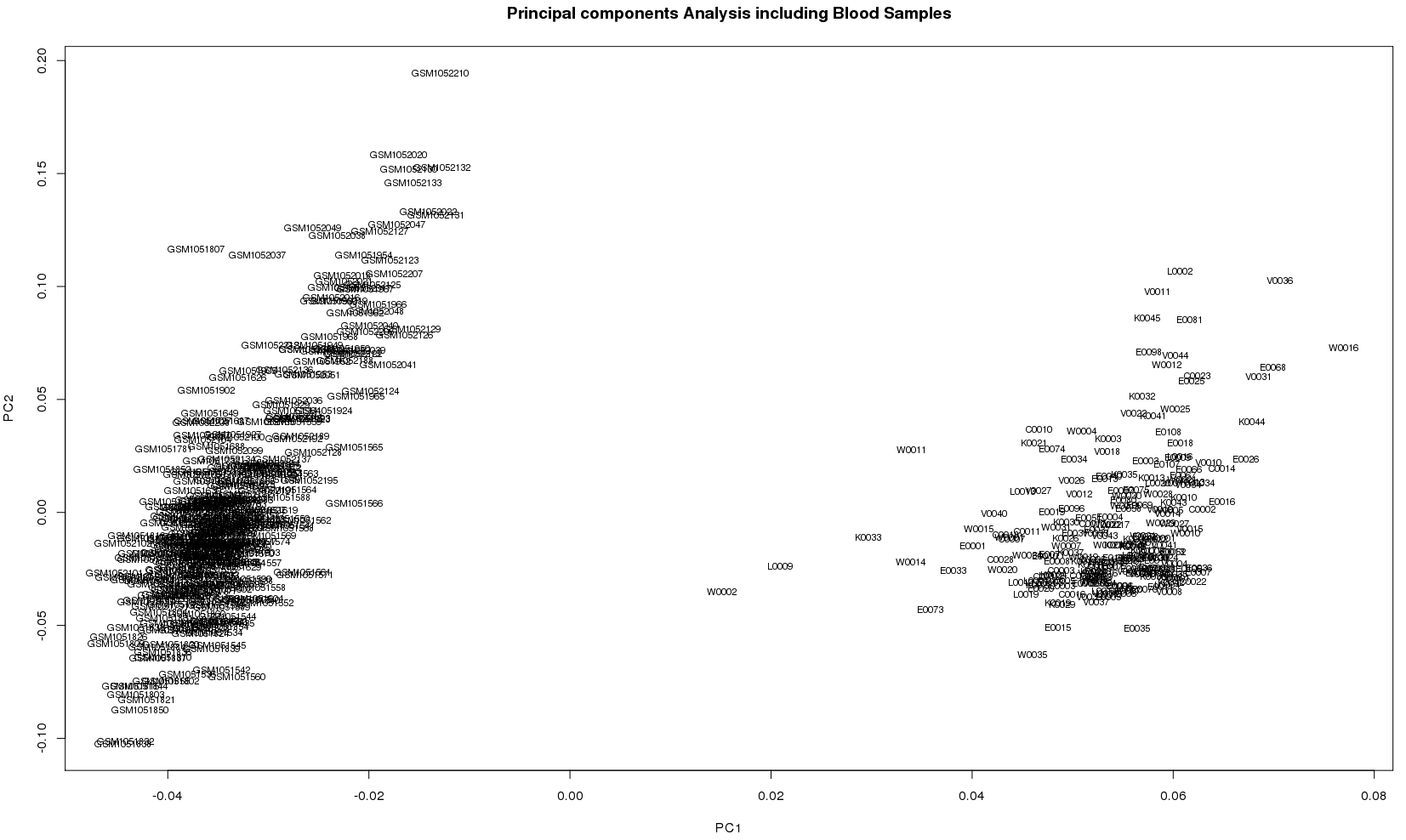
**

**Fig S2. Exploration of surrogate variables.** A. Scree plot of the percentage of variance represented by each surrogate variable generated from the entire dataset. B. Heatmap of R squared values from linear regression modeling of each SV with each known covariate. C. Heatmap of adjusted p-values from linear regression modeling of each SV with known covariates and genetic clusters (C1 to C4).

**A**


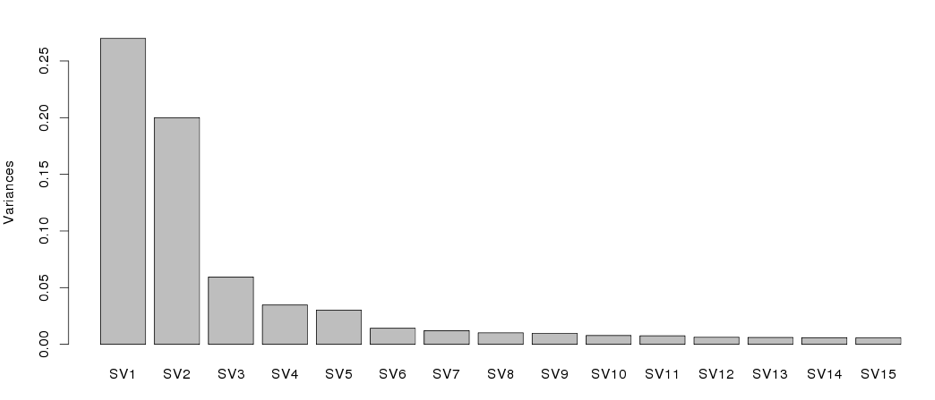


**
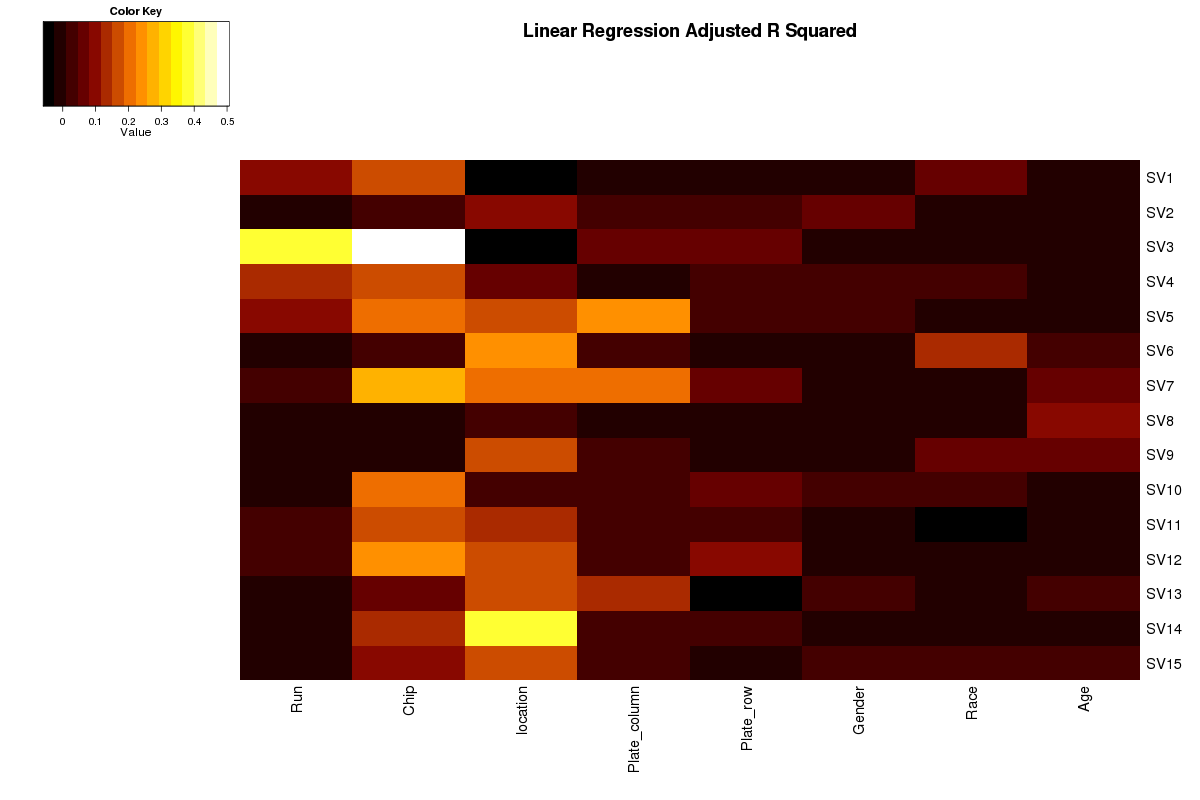
**

**B**

**
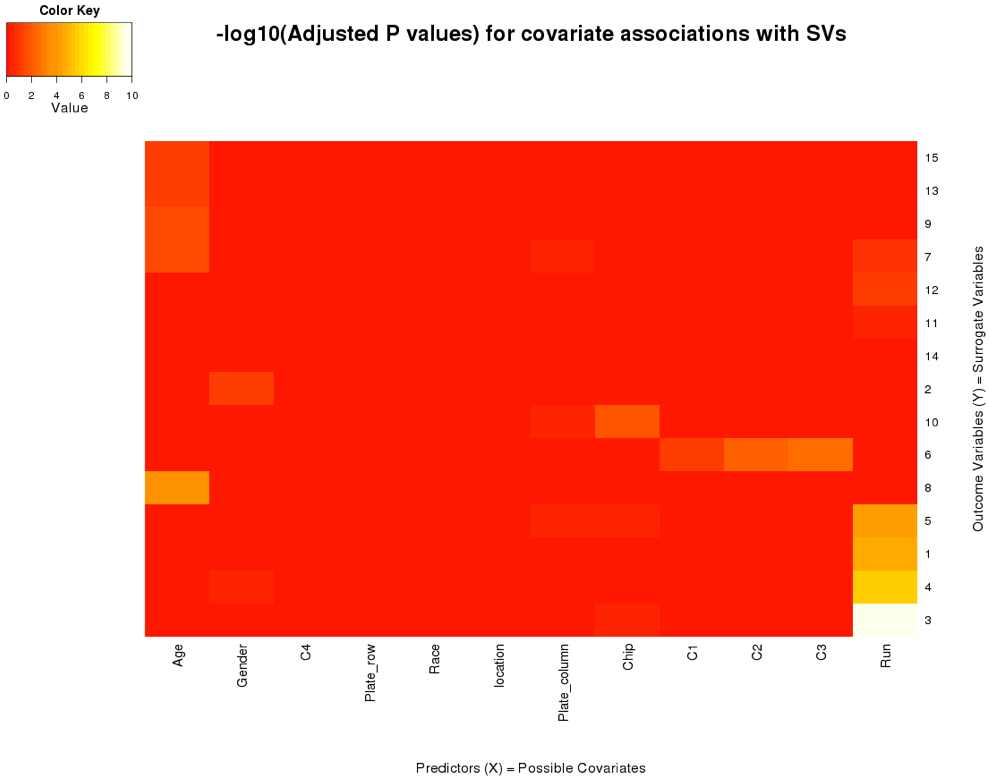
**

**C**

**Fig S3.** **Hierarchical clustering of individual based on MDS analysis of genotyping data.** Children with FASD (red) and controls (black) were clustered based on genetic scores from SNP genotyping. Two major subgroups were identified from the unsupervised hierarchical clustering.


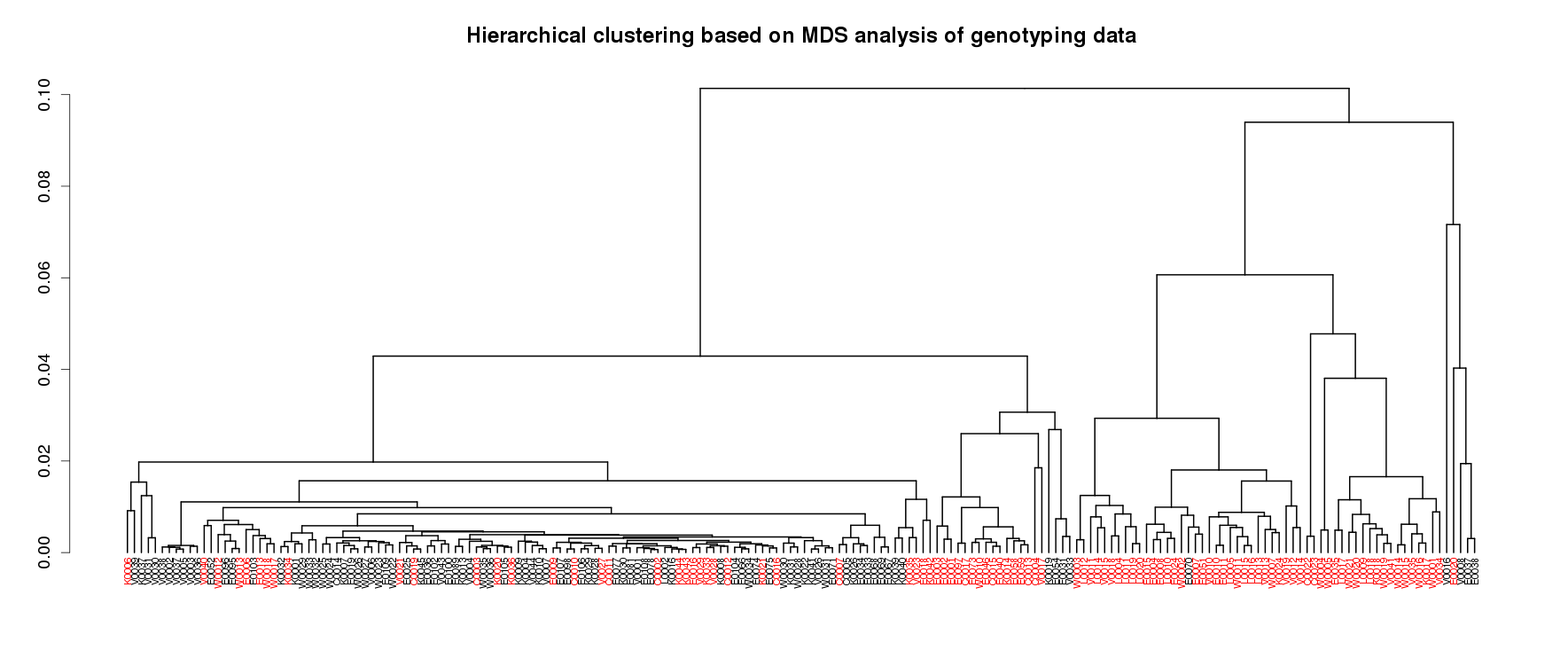


**Fig S4. Analysis of ethnically-biased probes in the 658 DM probes.** A. Distribution of “ethnic” rank in the significantly up- and down-methylated probes before and after ethnicity adjustment. B. ROC curve demonstrating that the “ethnic” probes are less able to predict the FASD DM probes after ethnicity adjustment.

**A**


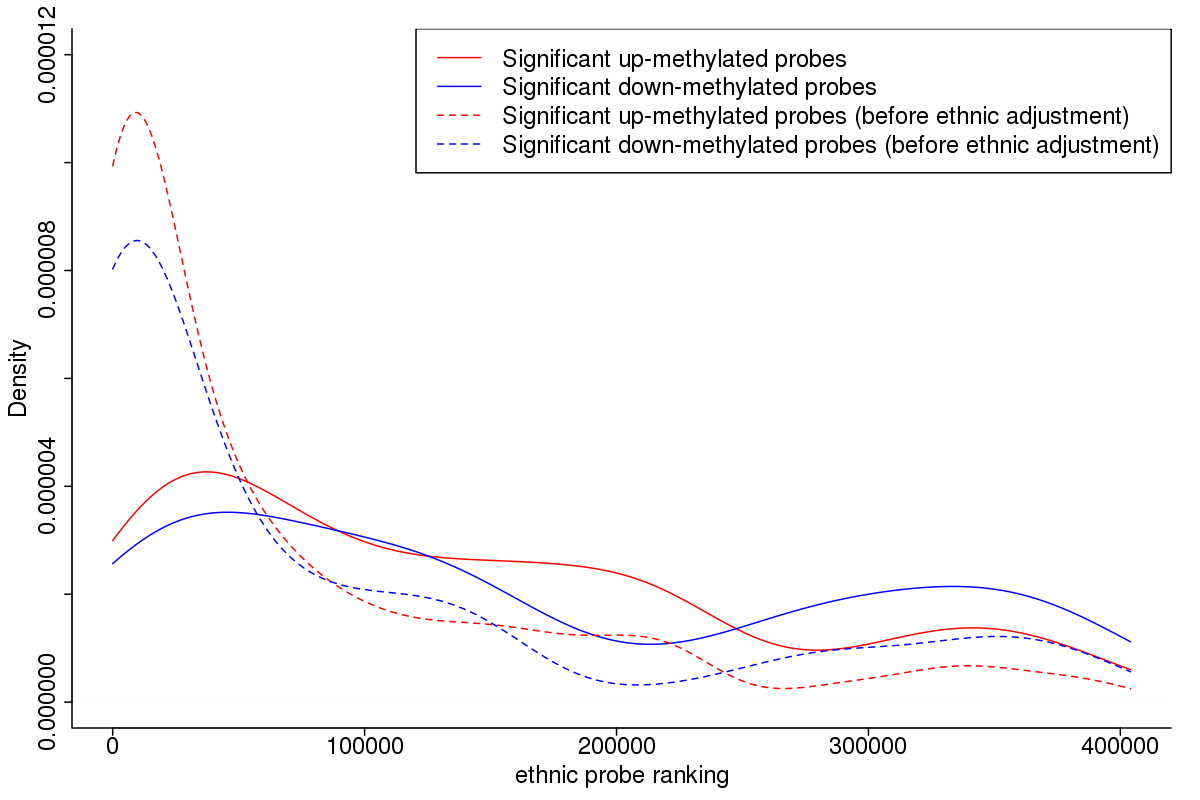


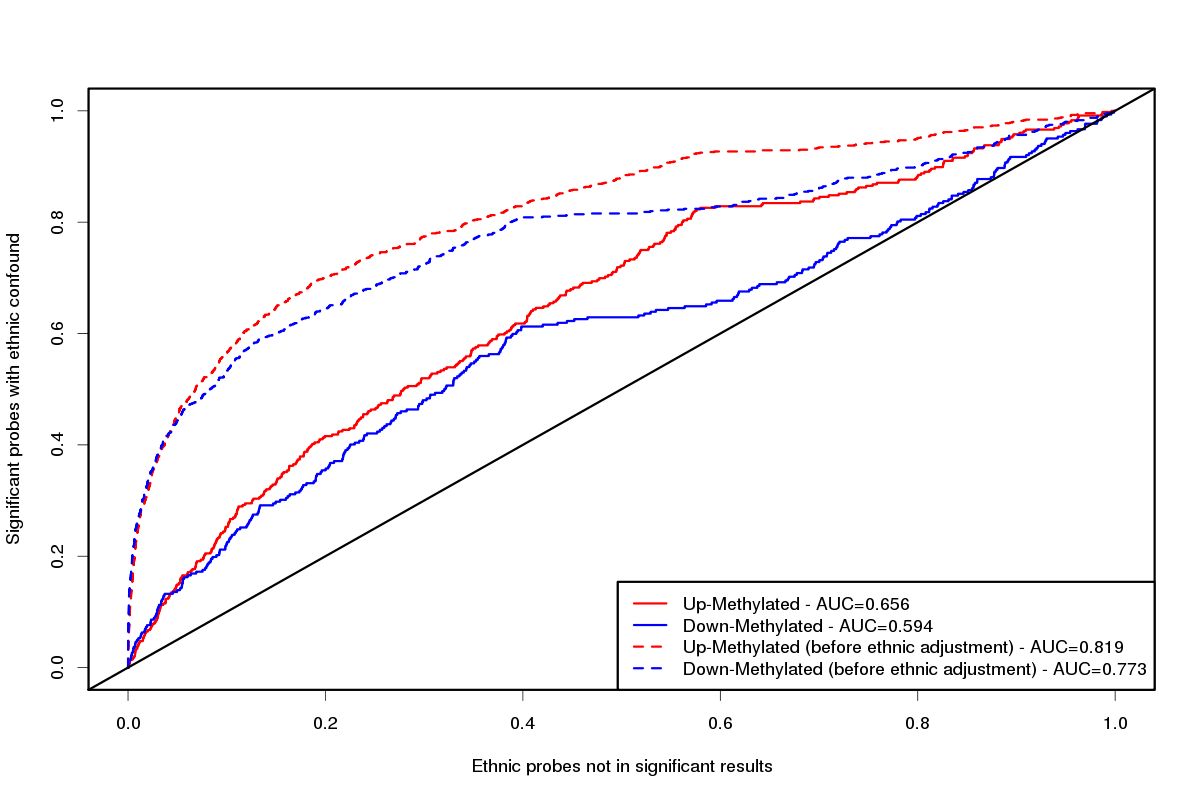


**B**

**Fig S5.** **Distribution of socio-economic status scores for children in the FASD and control groups. w**A. In the full dataset, the distribution of socio-economic status (SES) scores is significantly different between groups (Student’s t-test, p=0.00017). B. By contrast, in the more ethnically-homogeneous subgroup, the difference is no longer significant (Student’s t-test, p=0.16), suggesting that these effects may have been partially corrected through the method used for genetic background correction.

**
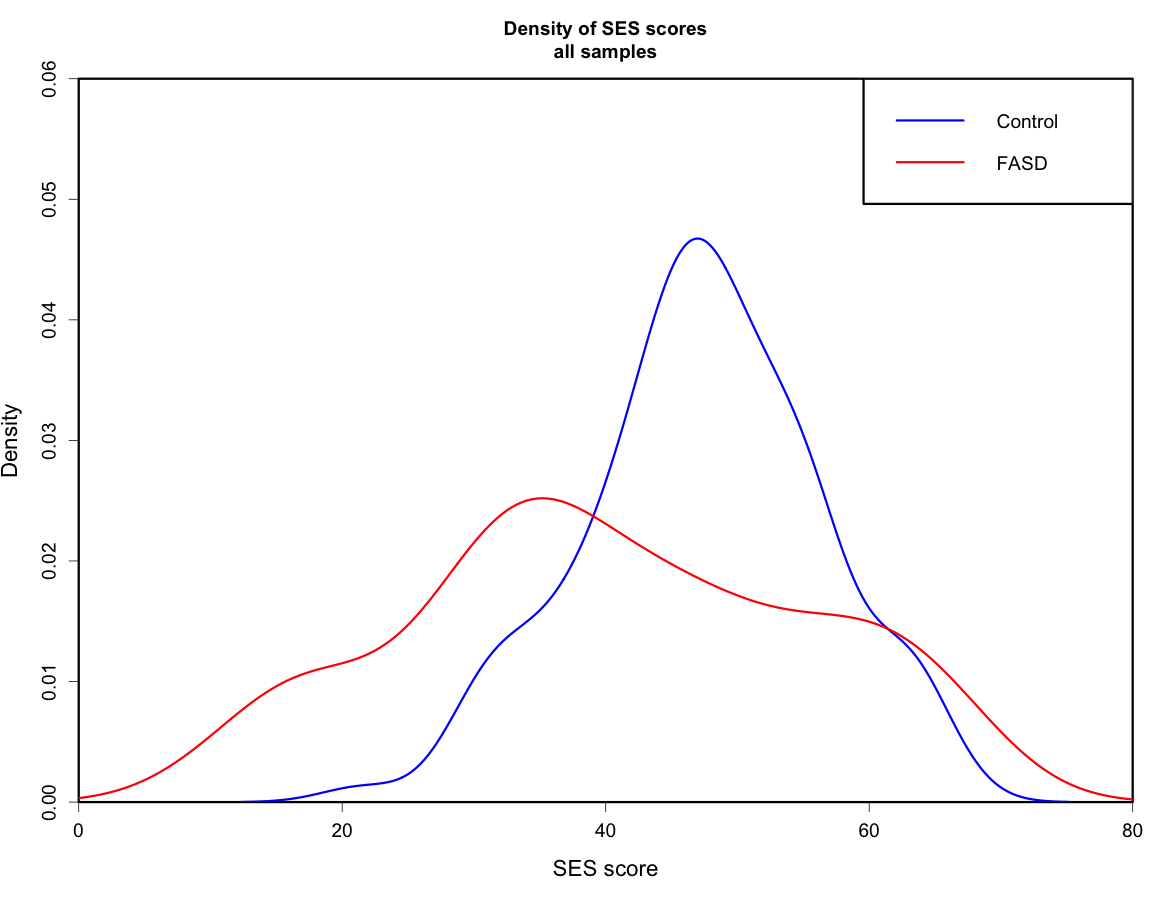

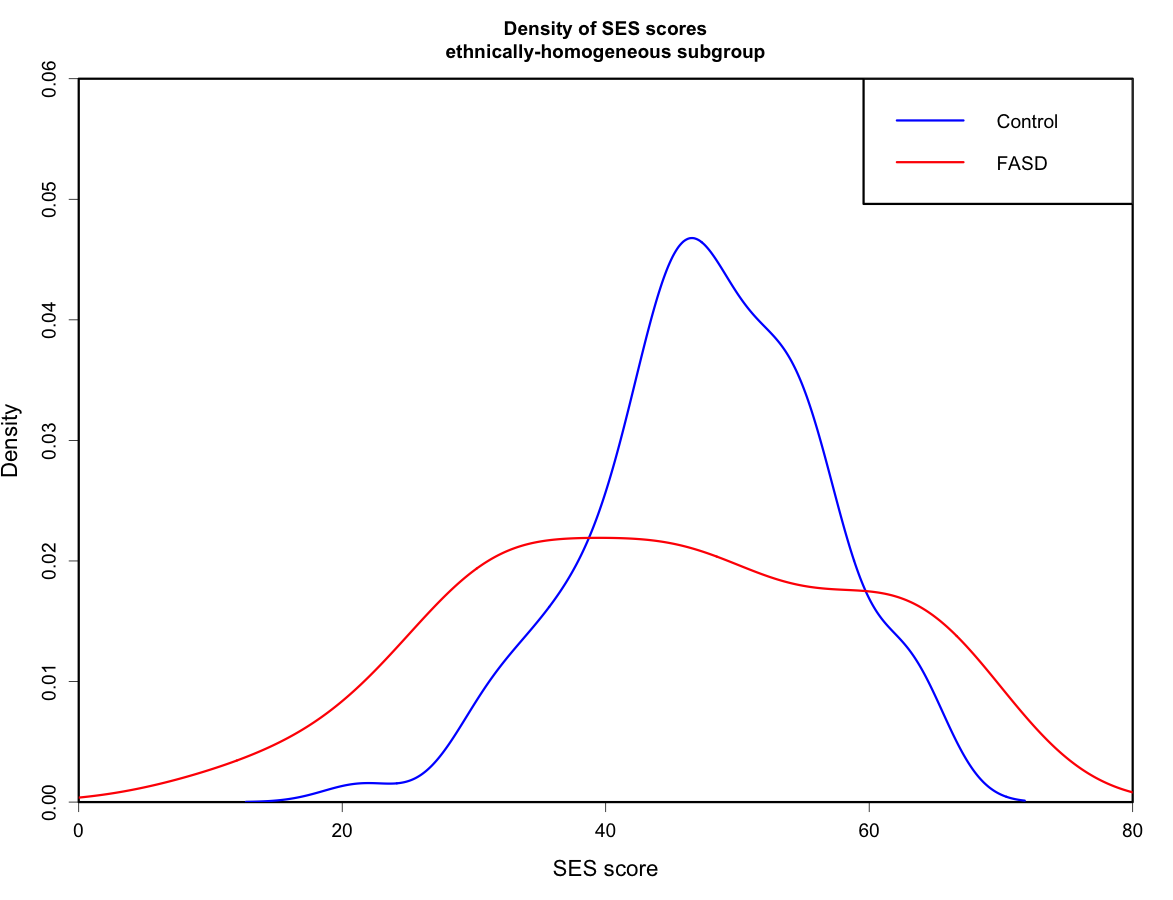
**

**B**

**A**

**Fig S6. Pyrosequencing verification.** Top row are Bland-Altman plots with the difference between the methylation values from the array and the pyrosequencing on the Y axis, and the average Beta values on the X axis. Bottom row are plots with the methylation level from the array on the Y axis and from the pyrosequencing on the X axis, with linear regression lines drawn as well as the Pearson correlation coefficient r.

**
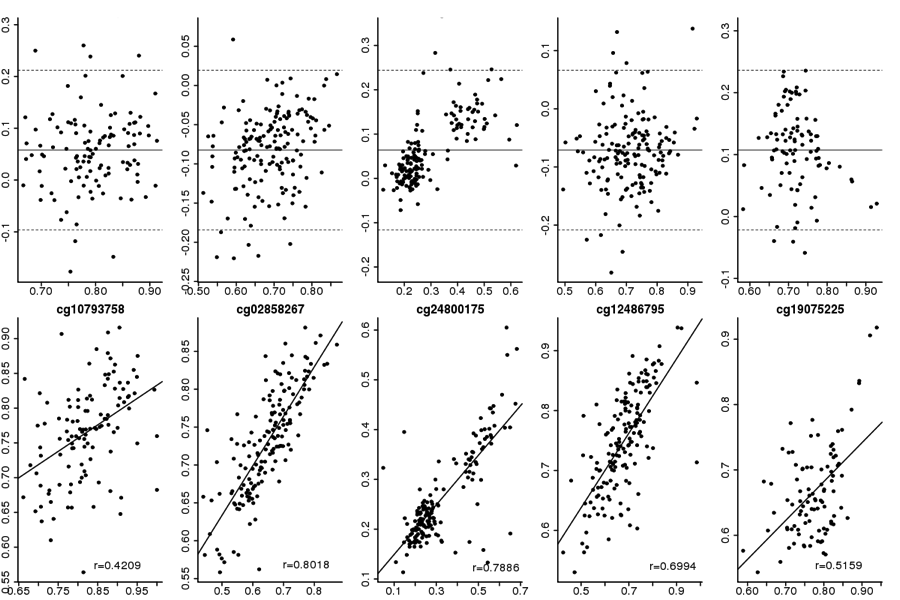
**

**Fig S7. Correlation of buccal and brain methylation at 658 DM CpGs.** Points represent an individual CpG methylation in brain samples (Farré et al. 2015, Epigenetics and Chromatin) and buccal epithelial cells. The overall spearman correlation of mean methylation between buccal and brain samples was 0.76.
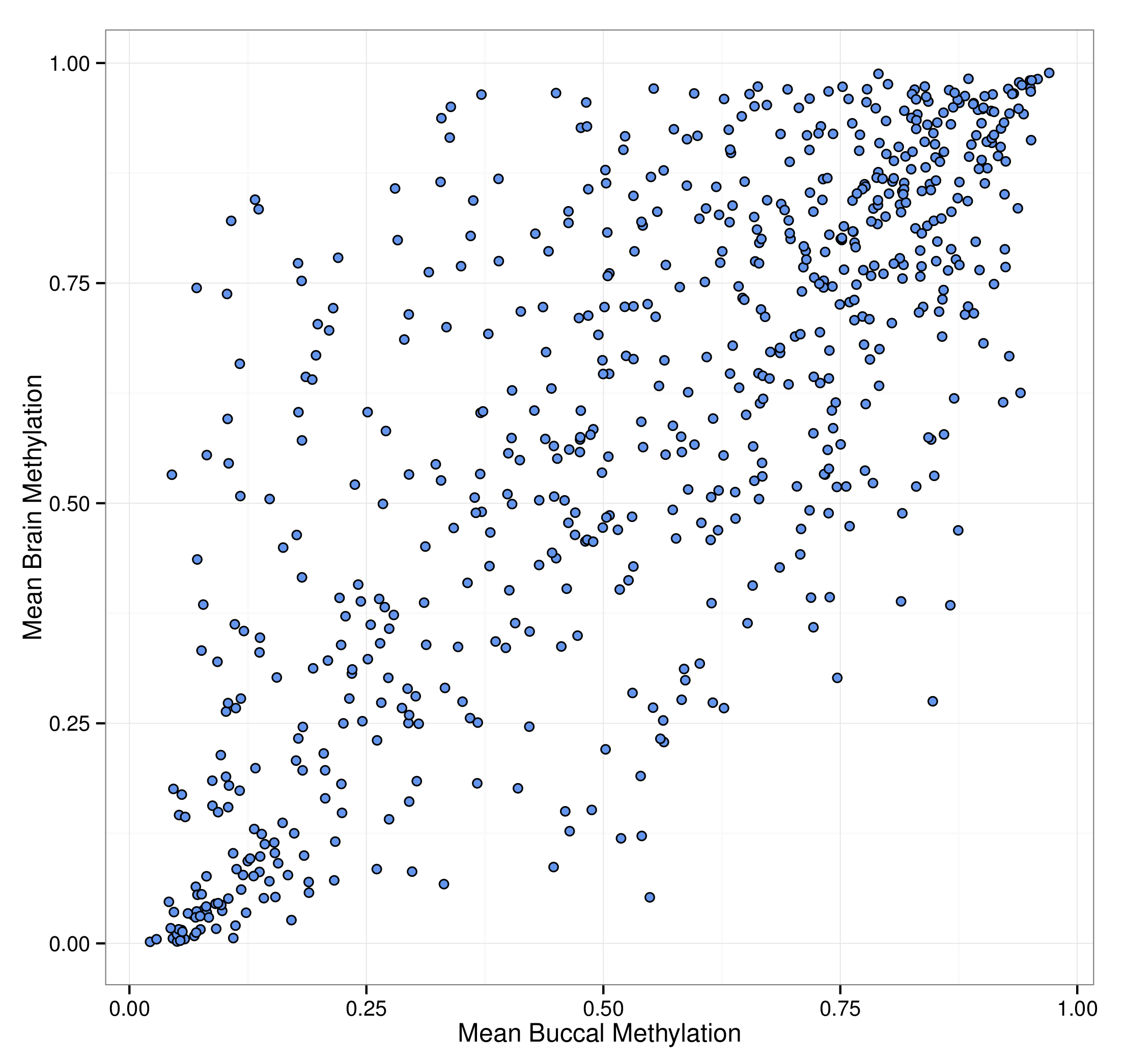


**Fig S8. Average distribution of DNA Methylation.** The distribution of average DNA methylation for all probes on the 450K array displayed a bimodal distribution, with probes clustering in the lowly (0-20%) and highly (80-100%) methylated ranges. By contrast, probes that were significantly differentially methylated in children with FASD versus controls were more prevalent in intermediately methylated regions (Student’s t test; p = 2.5e-09).


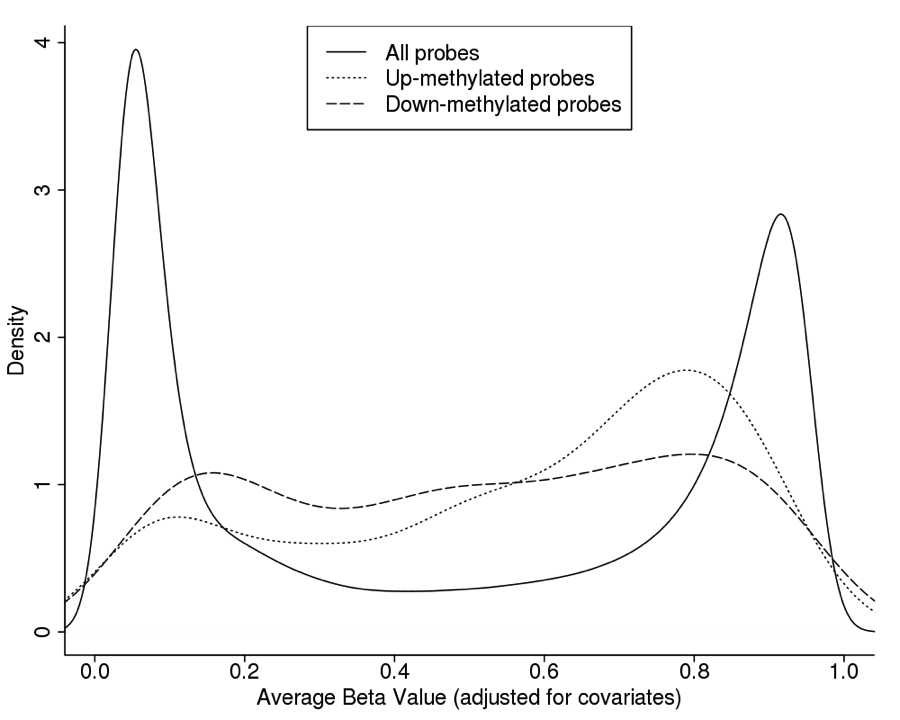


**Fig S9.** **P-value distributions from ANOVA analyses on 658 significant probes.** P-value distributions obtained from linear regression analyses were skewed to the left in controls versus both diagnosed FASD cases (FASDd) and children with PAE, but undiagnosed for FASD (PAE). By contrast, the p-value distribution comparing FASDd and PAE children revealed a relatively flat distribution, suggesting that there are little differences in DNA methylation patterns between diagnosed and undiagnosed children with PAE.

**
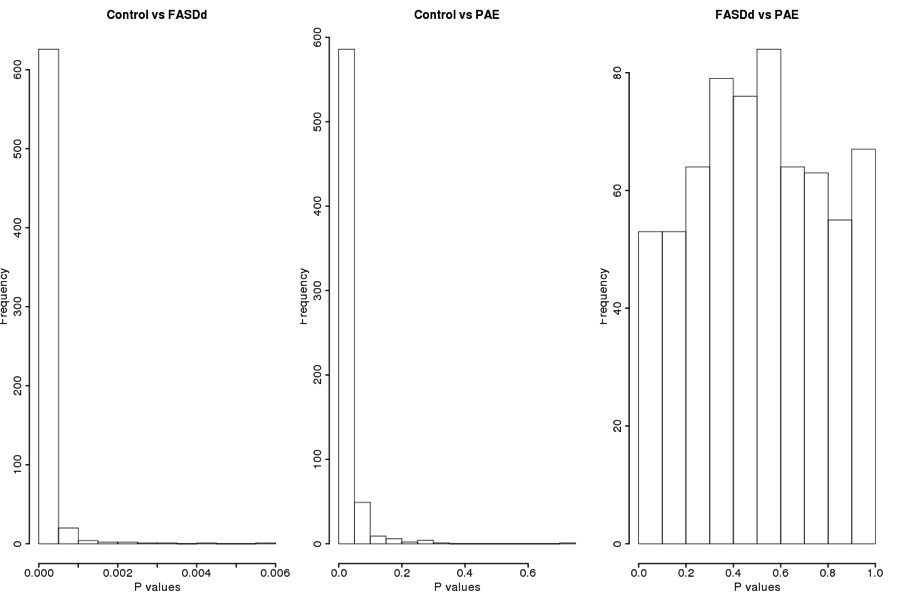
**
